# FRETBursts: An Open Source Toolkit for Analysis of Freely-Diffusing Single-Molecule FRET

**DOI:** 10.1371/journal.pone.0160716

**Published:** 2016-08-17

**Authors:** Antonino Ingargiola, Eitan Lerner, SangYoon Chung, Shimon Weiss, Xavier Michalet

**Affiliations:** Dept. Chemistry and Biochemistry, Univ. of California Los Angeles, Los Angeles, CA, United States of America; University of California Berkeley, UNITED STATES

## Abstract

Single-molecule Förster Resonance Energy Transfer (smFRET) allows probing intermolecular interactions and conformational changes in biomacromolecules, and represents an invaluable tool for studying cellular processes at the molecular scale. smFRET experiments can detect the distance between two fluorescent labels (donor and acceptor) in the 3-10 nm range. In the commonly employed confocal geometry, molecules are free to diffuse in solution. When a molecule traverses the excitation volume, it emits a burst of photons, which can be detected by single-photon avalanche diode (SPAD) detectors. The intensities of donor and acceptor fluorescence can then be related to the distance between the two fluorophores. While recent years have seen a growing number of contributions proposing improvements or new techniques in smFRET data analysis, rarely have those publications been accompanied by software implementation. In particular, despite the widespread application of smFRET, no complete software package for smFRET burst analysis is freely available to date. In this paper, we introduce FRETBursts, an open source software for analysis of freely-diffusing smFRET data. FRETBursts allows executing all the fundamental steps of smFRET bursts analysis using state-of-the-art as well as novel techniques, while providing an open, robust and well-documented implementation. Therefore, FRETBursts represents an ideal platform for comparison and development of new methods in burst analysis. We employ modern software engineering principles in order to minimize bugs and facilitate long-term maintainability. Furthermore, we place a strong focus on reproducibility by relying on Jupyter notebooks for FRETBursts execution. Notebooks are executable documents capturing all the steps of the analysis (including data files, input parameters, and results) and can be easily shared to replicate complete smFRET analyzes. Notebooks allow beginners to execute complex workflows and advanced users to customize the analysis for their own needs. By bundling analysis description, code and results in a single document, FRETBursts allows to seamless share analysis workflows and results, encourages reproducibility and facilitates collaboration among researchers in the single-molecule community.

## Introduction

### Open Science and Reproducibility

Over the past 20 years, single molecule FRET (smFRET) has grown into one of the most useful techniques in single-molecule spectroscopy [1, 2]. While it is possible to extract information on sub-populations using ensemble measurements (e.g. [3, 4]), smFRET unique feature is its ability to very straightforwardly resolve conformational changes of biomolecules or measure binding-unbinding kinetics in heterogeneous samples [5–9]. smFRET measurements on freely diffusing molecules (the focus of this paper) have the additional advantage, over measurements performed on immobilized molecules, of allowing to probe molecules and processes without perturbation from surface immobilization or additional functionalization needed for surface attachment [10, 11].

The increasing amount of work using freely-diffusing smFRET has motivated a growing number of theoretical contributions to the specific topic of data analysis [12–24]. Despite this profusion of publications, most research groups still rely on their own implementation of a limited number of methods, with very little collaboration or code sharing. To clarify this statement, let us point that our own group’s past smFRET papers merely mention the use of custom-made software without additional details [16, 17]. Even though some of these software tools are made available upon request, or sometimes shared publicly on websites, it remains hard to reproduce and validate results from different groups, let alone build upon them. Additionally, as new methods are proposed in literature, it is generally difficult to quantify their performance compared to other methods. An independent quantitative assessment would require a complete reimplementation, an effort few groups can afford. As a result, potentially useful analysis improvements are either rarely or slowly adopted by the community. In contrast with other established traditions such as sharing protocols and samples, in the domain of scientific software, we have relegated ourselves to islands of non-communication.

From a more general standpoint, the non-availability of the code used to produce scientific results, hinders reproducibility, makes it impossible to review and validate the software’s correctness and prevents improvements and extensions by other scientists. This situation, common in many disciplines, represents a real impediment to the scientific progress. Since the pioneering work of the Donoho group in the 90’s [25], it has become evident that developing and maintaining open source scientific software for reproducible research is a critical requirement of the modern scientific enterprise [26, 27].

Other disciplines have started tackling this issue [28], and even in the single-molecule field a few recent publications have provided software for analysis of surface-immobilized experiments [29–33]. For freely-diffusing smFRET experiments, although it is common to find mention of “code available from the authors upon reques” in publications, there is a dearth of such open source code, with, to our knowledge, the notable exception of a single example [34]. To address this issue, we have developed FRETBursts, an open source Python software for analysis of freely-diffusing single-molecule FRET measurements. FRETBursts can be used, inspected and modified by anyone interested in using state-of-the art smFRET analysis methods or implementing modifications or completely new techniques. FRETBursts therefore represents an ideal platform for quantitative comparison of different methods for smFRET burst analysis. Technically, a strong emphasis has been given to the reproducibility of complete analysis workflows. FRETBursts uses Jupyter Notebooks [35], an interactive and executable document containing textual narrative, input parameters, code, and computational results (tables, plots, etc.). A notebook thus captures the various analysis steps in a document which is easy to share and execute. To minimize the possibility of bugs being introduced inadvertently [36], we employ modern software engineering techniques such as unit testing and continuous integration [28, 37]. FRETBursts is hosted on GitHub [38, 39], where users can write comments, report issues or contribute code. In a related effort, we recently introduced Photon-HDF5 [40], an open file format for timestamp-based single-molecule fluorescence experiments. An other related open source tool is PyBroMo [41], a freely-diffusing smFRET simulator which produces Photon-HDF5 files that are directly analyzable with FRETBursts. Together with all the aforementioned tools, FRETBursts contributes to the growing ecosystem of open tools for reproducible science in the single-molecule field.

### Paper Overview

This paper is written as an introduction to smFRET burst analysis and its implementation in FRETBursts. The aim is illustrating the specificities and trade-offs involved in various approaches with sufficient details to enable readers to customize the analysis for their own needs.

After a brief overview of FRETBursts features (section FRETBursts Overview), we introduce essential concepts and terminology for smFRET burst analysis (section Architecture and Concepts). In section smFRET Burst Analysis, we illustrate the steps involved in smFRET burst analysis: (i) data loading (section Loading Data), (ii) definition of the excitation alternation periods (section Alternation Parameters), (iii) background correction (section Background Estimation), (iv) burst search (section Burst Search), (v) burst selection (section Burst Selection) and (vi) FRET histogram fitting (section Population Analysis). We conclude the section by surveying different methods proposed in litterature to study FRET dynamics (section FRET Dynamics). As an example of implementation of an advanced data processing technique, section Implementing Burst Variance Analysis walks the reader through implementing Burst Variance Analysis (BVA) [23]. Finally, section Conclusions summarizes what we believe to be the strengths of FRETBursts software.

Throughout this paper, links to relevant sections of documentation and other web resources are displayed as “(link)”. In order to make the text more legible, we have concentrated Python-specific details in paragraphs titled *Python details*. These subsections provide deeper insights for readers already familiar with Python and can be initially skipped by readers who are not. Finally, note that all commands and figures in this paper can be regenerated using the accompanying notebooks (link).

## FRETBursts Overview

### Technical Features

FRETBursts can analyze smFRET measurements from one or multiple excitation spots [42]. The supported excitation schemes include single laser, alternating laser excitation (ALEX) with either CW lasers (μs-ALEX [43]) or pulsed lasers (ns-ALEX [44] or pulsed-interleaved excitation (PIE) [45]).

The software implements both standard and novel algorithms for smFRET data analysis including background estimation as a function of time (including background accuracy metrics), sliding-window burst search [10], dual-channel burst search (DCBS) [17] and modular burst selection methods based on user-defined criteria (including a large set of pre-defined selection rules). Novel features include burst size selection with *γ*-corrected burst sizes, burst weighting, burst search with background-dependent threshold (in order to guarantee a minimal signal-to-background ratio [46]). Moreover, FRETBursts provides a large set of fitting options to characterize FRET subpopulations. In particular, distributions of burst quantities (such as *E* or *S*) can be assessed through (1) histogram fitting (with arbitrary model functions), (2) non-parametric weighted kernel density estimation (KDE), (3) weighted expectation-maximization (EM), (4) maximum likelihood fitting using Gaussian models or Poisson statistic. Finally FRETBursts includes a large number of predefined and customizable plot functions which (thanks to the *matplotlib* graphic library [47]) produce publication quality plots in a wide range of formats.

Additionally, implementations of population dynamics analysis such as Burst Variance Analysis (BVA) [23] and two-channel kernel density distribution estimator (2CDE) [24] are available as FRETBursts notebooks (BVA link, 2CDE link).

### Software Availability

FRETBursts is hosted and openly developed on GitHub. FRETBursts homepage (link) contains links to the various resources. Pre-built packages are provided for Windows, OS X and Linux. Installation instructions can be found in the Reference Documentation (link). A description of FRETBursts execution using Jupyter notebooks is reported in S1 Appendix. Detailed information on development style, testing strategies and contributions guidelines are reported in S2 Appendix. Finally, to facilitate evaluation and comparison with other software, we set up an on-line services allowing to execute FRETBursts without requiring any installation on the user’s computer (link).

## Architecture and Concepts

In this section, we introduce some general burst analysis concepts and notations used in FRETBursts.

### Photon Streams

The raw data collected during a smFRET experiment consists in one or more arrays of photon timestamps, whose temporal resolution is set by the acquisition hardware, typically between 10 and 50 ns. In single-spot measurements, all timestamps are stored in a single array. In multispot measurements [42], there are as many timestamps arrays as excitation spots. Each array contains timestamps from both donor (D) and acceptor (A) channels. When alternating excitation lasers are used (ALEX measurements) [16], a further distinction between photons emitted during the D or A excitation periods can be made.

In FRETBursts, the corresponding sets of photons are called “photon streams” and are specified with a Ph_sel object (link). In non-ALEX smFRET data, there are 3 photon streams (Table 1), while in two-color ALEX data, there are 5 streams (Table 2).

**Table 1:** Photon selection syntax (non-ALEX).

Photon selection	code
All-photons	Ph_sel(’all’)
D-emission	Ph_sel(Dex=’Dem’)
A-emission	Ph_sel(Dex=’Aem’)

**Table 2:** Photon selection syntax (ALEX).

Photon selection	code
All-photons	Ph_sel(’all’)
D-emission during D-excitation	Ph_sel(Dex=’Dem’)
A-emission during D-excitation	Ph_sel(Dex=’Aem’)
D-emission during A-excitation	Ph_sel(Aex=’Dem’)
A-emission during A-excitation	Ph_sel(Aex=’Aem’)

The Ph_sel class (link) allows the specification of any combination of photon streams. For example, in ALEX measurements, the D-emission during A-excitation stream is usually ignored because it does not contain any useful signal [16]. To indicate all but photons in this photon stream, the syntax is Ph_sel(Dex=’DAem’, Aex=’Aem’), which indicates selection of donor and acceptor photons (DAem) during donor excitation (Dex) and only acceptor photons (Aem) during acceptor excitation (Aex).

### Background Definitions

An estimation of the background rates is needed to both select a proper threshold for burst search, and to correct the raw burst counts by subtracting background counts.

**Fig 1:**
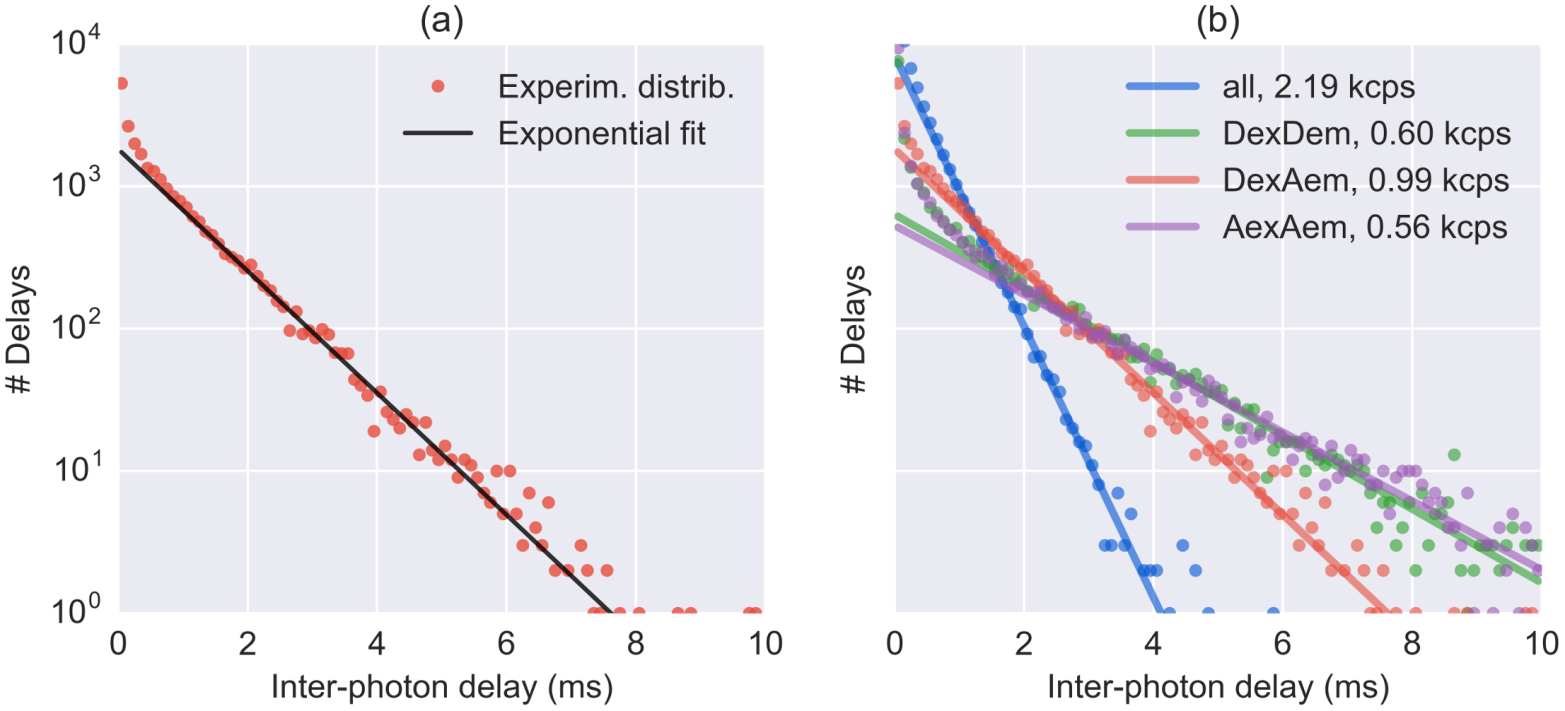
Inter-photon delays fitted with and exponential function. Experimental distributions of inter-photon delays (*dots*) and corresponding fits of the exponential tail (*solid lines*). (*Panel a*) An example of inter-photon delays distribution (*red dots*) and an exponential fit of the tail of the distribution (*black line*). (*Panel b*) Inter-photon delays distribution and exponential fit for different photon streams as obtained with dplot(d, hist_bg). The *dots* represent the experimental histogram for the different photon streams. The *solid lines* represent the corresponding exponential fit of the tail of the distributions. The legend shows abbreviations of the photon streams and the fitted background rates.

The recorded stream of timestamps is the result of two processes: one characterized by a high count rate, due to fluorescence photons of single molecules crossing the excitation volume, and another characterized by a lower count rate, due to “background counts” originating from detector dark counts, afterpulsing, out-of-focus molecules and sample scattering and/or impurities [20, 48]. The signature of these two types of processes can be observed in the inter-photon delays distribution (i.e. the waiting times between two subsequent timestamps) as illustrated in Fig 1(a). The “tail” of the distribution (a straight line in semi-log scale) corresponds to exponentially-distributed time-delays, indicating that those counts are generated by a Poisson process. At short timescales, the distribution departs from an exponential due to the contribution of the higher rate process of single molecules traversing the excitation volume. To estimate the background rate (i.e. the inverse of the exponential time constant), it is necessary to define a time-delay threshold above which the distribution can be considered exponential. Finally, a parameter estimation method needs to be specified, such as Maximum Likelihood Estimation (MLE) or non-linear least squares curve fitting of the time-delay histogram (both supported in FRETBursts).

**Fig 2:**
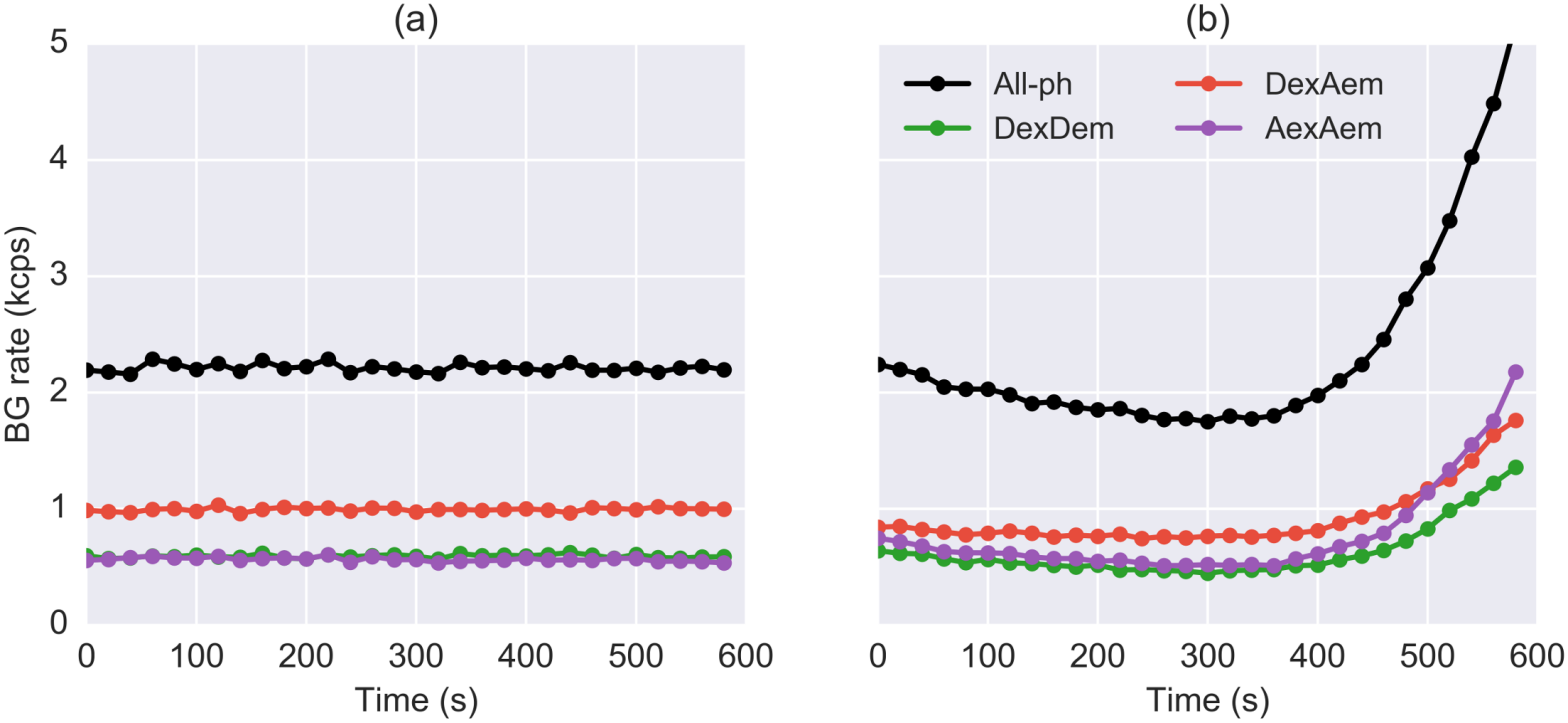
Background rates as a function of time. Estimated background rate as a function of time for two μs-ALEX measurements. Different colors represent different photon streams. (*Panel a*) A measurement performed with a sealed sample chamber exhibiting constant a background as a function of time. (*Panel b*) A measurement performed on an unsealed sample exhibiting significant background variations due to sample evaporation and/or photobleaching (likely impurities on the cover-glass). These plots are produced by the command dplot(d, timetrace_bg) after estimation of background. Each data point in these figures is computed for a 30 s time window.

It is advisable to monitor the background as a function of time throughout the measurement, in order to account for possible variations. Experimentally, we found that when the background is not constant, it usually varies on time scales of tens of seconds (see Fig 2). FRETBursts divides the acquisition in constant-duration time windows called *background periods* and computes the background rates for each of these windows (see section Background Estimation). Note that FRETBursts uses these local background rates also during burst search, in order to compute time-dependent burst detection thresholds and for background correction of burst data (see section Burst Search).

### The Data Class

The Data class (link) is the fundamental data container in FRETBursts. It contains the measurement data and parameters (attributes) as well as several methods for data analysis (background estimation, burst search, etc…). All analysis results (bursts data, estimated parameters) are also stored as Data attributes.

There are 3 important “burst counts” attributes which contain the number of photons detected in the donor or the acceptor channel during donor or acceptor excitation periods (Table 3). The attributes in Table 3 are background-corrected by default. Furthermore, na is corrected for leakage and direct excitation (section Bursts Corrections) if the relative coefficients are specified (by default they are set to 0). There is also a closely related attribute named nda for donor photons detected during acceptor excitation. nda is normally neglected as it only contains background.

**Table 3:** Data attributes names and descriptions for burst photon counts in different photon streams.

Name	Description
nd	number of photons detected by the donor channel (during donor excitation period in ALEX case)
na	number of photons detected by the acceptor channel (during donor excitation period in ALEX case)
naa	number of photons detected by the acceptor channel during acceptor excitation period (present only in ALEX measurements)

#### Python details

Many Data attributes are lists of arrays (or scalars) with the length of the lists equal to the number of excitation spots. This means that in single-spot measurements, an array of burst-data is accessed by specifying the index as 0, for example Data.nd[0]. Data implements a shortcut syntax to access the first element of a list with an underscore, so that an equivalently syntax is Data.nd_ instead of Data.nd[0].

### Introduction to Burst Search

Identifying single-molecule fluorescence bursts in the stream of photons is one of the most crucial steps in the analysis of freely-diffusing single-molecule FRET data. The widely used “sliding window” algorithm, introduced by the Seidel group in 1998 [10, 12], involves searching for *m* consecutive photons detected during a period shorter than Δ*t*. In other words, bursts are regions of the photon stream where the local rate (computed using *m* photons) is above a minimum threshold rate. Since a universal criterion to choose the rate threshold and the number of photons *m* is, as of today, lacking, it has become a common practice to manually adjust those parameters for each specific measurement. Commonly employed values for *m* are between 5 and 15 photons.

A more general approach consists in taking into account the background rate of the specific measurements and in choosing a rate threshold that is *F* times larger than the background rate (typical values for *F* are between 4 and 9). This approach ensures that all resulting bursts have a signal-to-background ratio (SBR) larger than (*F* − 1) [46]. A consistent criterion for choosing the threshold is particularly important when comparing different measurements with different background rates, when the background significantly varies during measurements or in multi-spot measurements where each spot has a different background rate.

A second important aspect of burst search is the choice of photon stream used to perform the search. In most cases, for instance when identifying FRET sub-populations, the burst search should use all photons, the so called all-photon burst search (APBS) [10, 12, 17]. In other cases, for example when focusing on donor-only or acceptor-only populations, it is better to perform the search using only donor or acceptor signal. In order to handle the general case and to provide flexibility, FRETBursts allows performing the burst search on arbitrary selections of photons (see section Photon Streams for more information on photon stream definitions).

Additionally, Nir *et al.* [17] proposed a dual-channel burst search (DCBS) which can help mitigating artifacts due to photophysics effects such as blinking. During DCBS, a search is performed on two photon streams and bursts are defined as periods during which both photon streams exhibit a rate higher than the threshold, implementing the equivalent of an AND logic operation. Conventionally, the term DCBS refers to a burst search where the two photon streams are (1) all photons during donor excitation (Ph_sel(Dex=’DAem’)) and (2) acceptor channel photons during acceptor excitation (Ph_sel(Aex=’Aem’)). In FRETBursts, the user can choose arbitrary photon streams as input, an in general this kind of search is called a “AND-gate burst search”. For additional details on burst search refer to the documentation (link).

After burst search, it is necessary to further select bursts, for instance by specifying a minimum number of photons (or burst size). In the most basic form, this selection can be performed during burst search by discarding bursts with size smaller than a threshold *L* (typically 30 or higher), as originally proposed by Eggeling *et al.* [10]. This method, however, neglects the effect of background and *γ* factor on the burst size and can lead to a selection bias for some channels and/or sub-populations. For this reason, we suggest performing a burst size selection after background correction, taking into account the *γ* factor, as discussed in sections Corrected Burst Sizes and Weights and Burst Selection. In special cases, users may choose to replace (or combine) the burst selection based on burst size with another criterion such as burst duration or brightness (see section Burst Selection).

### Corrected Burst Sizes and Weights

The number of photons detected during a burst –the “burst size”– is computed using either all photons, or photons detected during donor excitation period. To compute the burst size, FRETBursts uses one of the following formulas:

$$\begin{array}{c} n_{dex}=n_{a}+\gamma \;n_{d} \end{array}$$ (1)

$$\begin{array}{c} n_{t}=n_{a}+\gamma \;n_{d}+n_{aa} \end{array}$$ (2)

where *n*_*d*_, *n*_*a*_ and *n*_*aa*_ are, similarly to the attributes in Table 3, the background-corrected burst counts in different channels and excitation periods. The factor *γ* takes into account different fluorescence quantum yields of donor and acceptor fluorophores and different photon detection efficiencies between donor and acceptor detection channels [16, 49]. Eq (1) includes counts collected during donor excitation periods only, while Eq (2) includes all counts. Burst sizes computed according to Eqs (1) or (2) are called *γ*-corrected burst sizes.

The burst search algorithm yields a set of bursts whose sizes approximately follow an exponential distribution. Compared to bursts with smaller sizes, bursts with large sizes are less frequent, but contain more information per-burst (having higher SNR). Therefore, selecting bursts by size is an important step (see Burst Selection). A threshold set too low may result in unresolvable sub-populations because of broadening of FRET peaks and appearance of shot-noise artifacts in the FRET (and *S*) distribution (i.e. spurious narrow peaks due to *E* and *S* being computed as the ratio of small integers). Conversely, too large a threshold may result in too low a number of bursts therefore poor representation of the FRET distribution. Additionally, especially when computing fractions of sub-populations (e.g. ratio of number of bursts in each sub-population), it is important to use *γ*-corrected burst sizes as selection criterion, in order to avoid under-representing some FRET sub-populations due to different quantum yields of donor and acceptor dyes and/or different photon detection efficiencies of donor and acceptor channels.

An alternative method to apply *γ* correction consist in discarding a constant fraction of photons chosen randomly from either the D_em_ or A_em_ photon stream [17]. This simple method transforms the measurement data in order to achieve *γ* = 1, overcoming the issue of selection bias between populations. This approach has also the advantage of preserving the binomial distribution of D and A photons in each burst, so that peaks of FRET populations are easier to model statistically. The only drawback is that, by discarding a fraction of photons, this method leads to information loss and therefore to a potential decrease in sensitivity and/or accuracy.

A simple way to mitigate the dependence of the FRET distribution on the burst size selection threshold is weighting bursts proportionally to their size so that the bursts with largest sizes will have the largest weights. Using size as weights (instead of any other monotonically increasing function of size) can be justified noticing that the variance of bursts proximity ratio (PR) is inversely proportional to the burst size (see S6 Appendix for details).

In general, a weighting scheme is used for building efficient estimators for a population parameter (e.g. the population FRET efficiency *E*_*p*_). But, it can also be used to build weighted histograms or Kernel Density Estimation (KDE) plots which emphasize FRET subpopulations peaks without excluding small size bursts. Traditionally, for optimal results when not using weights, the FRET histogram is manually adjusted by finding an ad-hoc (high) size-threshold which selects only bursts with the highest size (and thus lowest variance). Building size-weighted FRET histograms is a simple method to balance the need of reducing the peaks width with the need of including as much bursts as possible to reduce statistical noise. As a practical example, by fixing the burst size threshold to a low value (e.g. 10-20 photons) and using weights, is possible to build a FRET histogram with well-defined FRET sub-populations peaks without the need of searching an optimal burst-size threshold (S6 Appendix).

#### Python details

FRETBursts has the option to weight bursts using *γ*-corrected burst sizes which optionally include acceptor excitation photons naa. A weight proportional to the burst size is applied by passing the argument weights=’size’ to histogram or KDE plot functions. The weights keyword can be also passed to fitting functions in order to fit the weighted E or S distributions (see section Population Analysis). Other weighting functions (for example depending quadratically on the size) are listed in the fret_fit.get_weights documentation (link). However, using weights different from the size is not recommended due to their less efficient use of burst information (S6 Appendix).

## smFRET Burst Analysis

### Loading Data

While FRETBursts can load several data files formats, we encourage users to adopt the recently introduced Photon-HDF5 file format [40]. Photon-HDF5 is an HDF5-based, open format, specifically designed for freely-diffusing smFRET and other timestamp-based experiments. Photon-HDF5 is a self-documented, platform- and language-independent binary format, which supports compression and allows saving photon data (e.g. timestamps) and measurement-specific metadata (e.g. setup and sample information, authors, provenance, etc.). Moreover, Photon-HDF5 is designed for long-term data preservation and aims to facilitate data sharing between different software and research groups. All example data files provided with FRETBursts use the Photon-HDF5 format.

To load data from a Photon-HDF5 file, we use the function loader.photon_hdf5 (link):

d = loader.photon_hdf5(filename)

where filename is a string containing the file path. This command loads the measurement data into the variable d, a Data object (see section The Data Class).

The same command can load data from a variety of smFRET measurements supported by the Photon-HDF5 format, taking advantage of the rich metadata included with each file. For instance, data generated using different excitation schemes such as CW excitation or pulsed excitation, single-laser vs two alternating lasers, etc., or with any number of excitation spots, are automatically recognized and interpreted accordingly.

FRETBursts also supports loading μs-ALEX data stored in.sm files (a custom binary format used in the Weiss lab) and ns-ALEX data stored in.spc files (a binary format used by TCSPC Becker & Hickl acquisition hardware). Alternatively, these and other formats (such as ht3, a binary format used by PicoQuant hardware) can be converted into Photon-HDF5 files using phconvert, a file conversion library and utility for Photon-HDF5 (link). More information on loading different file formats can be found in the loader module’s documentation (link).

### Alternation Parameters

**Fig 3:**
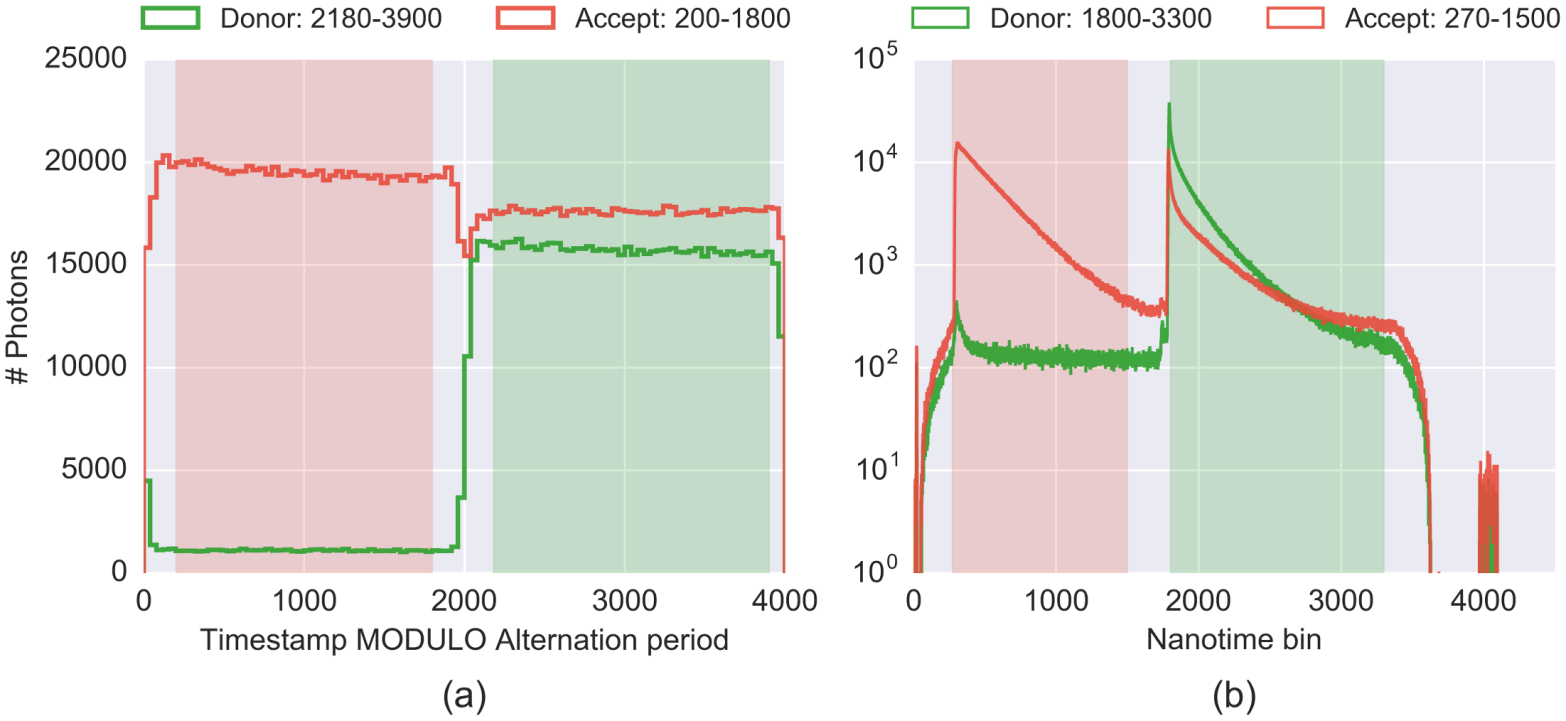
Alternation histograms for μs-ALEX and ns-ALEX measurements. Histograms used for the selection/determination of the alternation periods for two typical smFRET-ALEX experiments. Distributions of photons detected by donor channel are in *green*, and by acceptor channel in *red*. The light *green* and *red* shaded areas indicate the donor and acceptor period definitions. (a) μs-ALEX alternation histogram, i.e. histogram of timestamps *modulo* the alternation period for a smFRET measurement (in timestamp clock unit). (b) ns-ALEX TCSPC nanotime histogram for a smFRET measurement (in TDC or TAC bin unit). Both plots have been generated by the same plot function (plot_alternation_hist()). Additional information on these specific measurements can be found in the attached notebook (link).

For μs-ALEX and ns-ALEX data, Photon-HDF5 normally stores parameters defining alternation periods corresponding to donor and acceptor laser excitation. At load time, a user can plot these parameters and change them if deemed necessary. In μs-ALEX measurements [50], CW laser lines are alternated on timescales of the order of 10 to 100 μs. Plotting an histogram of timestamps modulo the alternation period, it is possible to identify the donor and acceptor excitation periods (see Fig 3a). In ns-ALEX measurements [44], pulsed lasers with equal repetition rates are delayed with respect to one another with typical delays of 10 to 100 ns. In this case, forming an histogram of TCSPC times (nanotimes) will allow the definition of periods of fluorescence after excitation of either the donor or the acceptor (see Fig 3b). In both cases, the function plot_alternation_hist (link) will plot the relevant alternation histogram (Fig 3) using currently selected (or default) values for donor and acceptor excitation periods.

To change the period definitions, we can type:

d.add(D_ON = (2100, 3900), A_ON = (100, 1900))

where D_ON and A_ON are pairs of numbers (tuples in python) representing the *start* and *stop* values for D or A excitation periods. The previous command works for both μs-ALEX and ns-ALEX measurements. After changing the parameters, a new alternation plot will show the updated period definitions.

The alternation period definition can be applied to the data using the function loader.alex_apply_period (link):

loader.alex_apply_period(d)

After this command, d will contain only photons inside the defined excitation periods. If the user needs to update the periods definition, the data file will need to be reloaded and the steps above repeated as described.

### Background Estimation

The first step of smFRET analysis involves estimating background rates. For example, the following command:

d.calc_bg(bg.exp_fit, time_s = 30, tail_min_us=’auto’)

estimates the background rates in windows of 30 s using the default iterative algorithm for choosing the fitting threshold (Background Definitions). Beginner users can simply use the previous command and proceed to burst search (Burst Search). For more advanced users, this section provides details on the different background estimation and plotting functions provided by FRETBursts.

First, we show how to estimate the background every 30 s, using a fixed inter-photon delay threshold of 2 ms (the same for all photon streams):

d.calc_bg(bg.exp_fit, time_s = 30, tail_min_us = 2000)

The first argument (bg.exp_fit) is the function used to fit the background rate for each photon stream (see section Background Definitions). The function bg.exp_fit estimates the background using a maximum likelihood estimation (MLE) of the delays distribution. The second argument, time_s, is the duration of the *background period* (section Background Definitions) and the third, tail_min_us, is the minimum inter-photon delay to use when fitting the distribution to the specified model function. To use different thresholds for each photon stream we pass a tuple (i.e. a comma-separated list of values, link) instead of a scalar. However, the recommended approach is to choose the threshold automatically using tail_min_us=’auto’. This approach uses an heuristic algorithm described in the *Background estimation* section of the μs-ALEX tutorial (link). Finally, it is possible to use a rigorous but slower approach to find an optimal threshold, as described in S5 Appendix.

FRETBursts provides two kinds of plots to represent the background. One shows the histograms of inter-photon delays compared to the fitted exponential distribution, shown in Fig 1) (see section Background Definitions for details on the inter-photon distribution). This plot is created with the command:

dplot(d, hist_bg, period = 0)

This command illustrates the general form of a plotting commands in FRETBursts, as described in S4 Appendix. Here we only note that the argument period is an integer specifying the background period to be plotted (when omitted, the default is 0, i.e. the first period). Fig 1 allows to quickly identify pathological cases where the background fitting procedure returns unreasonable values.

The second background-related plot represents a timetrace of background rates, as shown in Fig 2. This plot allows monitoring background rate variations occurring during the measurement and is obtained with the command:

dplot(d, timetrace_bg)

Normally, samples should have a fairly constant background rate as a function of time as in Fig 2(a). However, sometimes, non-ideal experimental conditions can yield a time-varying background rate, as illustrated in Fig 2(b). A possible reason for the observed behavior could be buffer evaporation from an open sample (we strongly recommend using a sealed observation chamber whenever possible). Additionally, cover-glass impurities can contribute to the background. These impurities tend to bleach on timescales of minutes resulting in background variations during the course of the measurement.

#### Python details

The estimated background rates are stored in the Data attributes bg_dd, bg_ad and bg_aa, corresponding to photon streams Ph_sel(Dex=’Dem’), Ph_sel(Dex=’Aem’) and Ph_sel(Aex=’Aem’) respectively. These attributes are lists of arrays (one array per excitation spot). The arrays contain the estimated background rates in the different time windows (background periods). Additional background fitting functions (e.g. least-square fitting of inter-photon delay histogram) are available in bg namespace (i.e. the background module, link).

### Burst Search

Following background estimation, burst search is the next step of the analysis. In FRETBursts, a standard burst search using a single photon stream (see section Introduction to Burst Search) is performed by calling the Data.burst_search method (link). For example, the following command:

d.burst_search(F = 6, m = 10, ph_sel = Ph_sel(’all’))

performs a burst search on all photons (ph_sel = Ph_sel(’all’)), with a count rate threshold equal to 6 times the local background rate (F = 6), using 10 consecutive photons to compute the local count rate (m = 10). A different photon stream, threshold (*F*) or number of photons *m* can be selected by passing different values. These parameters are good general-purpose starting point for smFRET analysis but they can be adjusted if needed.

Note that the previous burst search does not perform any burst size selection (however, by definition, the minimum bursts size is effectively *m*). An additional parameter *L* can be passed to impose a minimum burst size before any correction. However, it is recommended to select bursts only after applying background corrections, as discussed in the next section Burst Selection.

It might sometimes be useful to specify a fixed photon-rate threshold, instead of a threshold depending on the background rate, as in the previous example. In this case, instead of *F*, the argument min_rate_cps can be used to specify the threshold (in counts-per-second). For example, a burst search with a 50 kcps threshold is performed as follows:

d.burst_search(min_rate_cps = 50e3, m = 10,

      ph_sel = Ph_sel(’all’))

Finally, to perform a DCBS burst search (or in general an AND gate burst search, see section Introduction to Burst Search) we use the function burst_search_and_gate (link), as illustrated in the following example:

d_dcbs = bext.burst_search_and_gate(d, F = 6, m = 10)

The last command puts the burst search results in a new copy of the Data variable d (in this example the copy is called d_dcbs). Since FRETBursts shares the timestamps and detectors arrays between different copies of Data objects, the memory usage is minimized, even when several copies are created.

#### Python details

Note that, while d.burst_search() is a method of Data, bext.burst_search_and_gate() is a function in the bext module taking a Data object as a first argument and returning a new Data object.

The function burst_search_and_gate accepts optional arguments, ph_sel1 and ph_sel2, whose default values correspond to the classical DCBS photon stream selection (see section Introduction to Burst Search). These arguments can be specified to select different photon streams than those used in a classical DCBS.

The bext module (link) collects “plugin” functions that provides additional algorithms for processing Data objects.

### Bursts Corrections

In μs-ALEX, there are 3 important correction parameters: *γ*-factor, donor leakage into the acceptor channel and acceptor direct excitation by the donor excitation laser [16]. These corrections can be applied to burst data by simply assigning values to the respective Data attributes:

d.gamma = 0.85

d.leakage = 0.15

d.dir_ex = 0.08

These attributes can be assigned either before or after the burst search. In the latter case, existing burst data are automatically updated using the new correction parameters.

These correction factors can be used to display corrected FRET distributions. However, when the goal is to fit the FRET efficiency of sub-populations, it is simpler to fit the background-corrected PR histogram and then correct the population-level PR value (see SI in [16]). Correcting PR of each population (instead of correcting the data in each burst) avoids distortion of the FRET distribution and keeps peaks of static FRET subpopulations closer to the ideal binomial statistics [19].

FRETBursts implements the correction formulas for *E* and *S* in the functions fretmath.correct_E_gamma_leak_dir and fretmath.correct_S (link). A derivation of these correction formulas (using computer-assisted algebra) can be found online as an interactive notebook (link).

### Burst Selection

After burst search, it is common to select bursts according to different criteria. One of the most common is burst size.

For instance, to select bursts with more than 30 photons detected during the donor excitation (computed after background correction), we use following command:

ds = d.select_bursts(select_bursts.size, th1 = 30)

The previous command creates a new Data variable (ds) containing the selected bursts. th1 defines the lower bound for burst size, while th2 defines the upper bound (when not specified, as in the previous example, the upper bound is +∞). As before, the new object (ds) will share the photon data arrays with the original object (d) in order to minimize the amount of used memory.

The first argument of select_bursts (link) is a python function implementing the “selection rule” (select_bursts.size in this example); all remaining arguments (only th1 in this case) are parameters of the selection rule. The select_bursts module (link) contains numerous built-in selection functions (link). For example, select_bursts.ES is used to select a region on the E-S ALEX histogram, select_bursts.width to select bursts based on their duration. New custom criteria can be readily implemented by defining a new selection function, which requires only a couple of lines of code (see the select_bursts module’s source code for examples, link).

Finally, different criteria can be combined sequentially. For example, with the following commands:

ds = d.select_bursts(select_bursts.size,

         th1 = 50, th2 = 200)

dsw = ds.select_bursts(select_bursts.width,

         th1 = 0.5e-3, th2 = 3e-3)

bursts in dsw will have sizes between 50 and 200 photons, and duration between 0.5 and 3 ms.

#### Burst Size Selection

In the previous section, we selected bursts by size, using only photons detected in both D and A channels during D excitation (i.e. D_ex_ photons), as in Eq (1). Alternatively, a threshold on the burst size computed including all photons can be applied by adding *n*_*aa*_ to the burst size (see Eq (2)). This is achieved by passing add_naa = True to the selection function. The complete selection command is:

ds = d.select_bursts(select_bursts.size,

       th1 = 30, add_naa = True)

**Fig 4:**
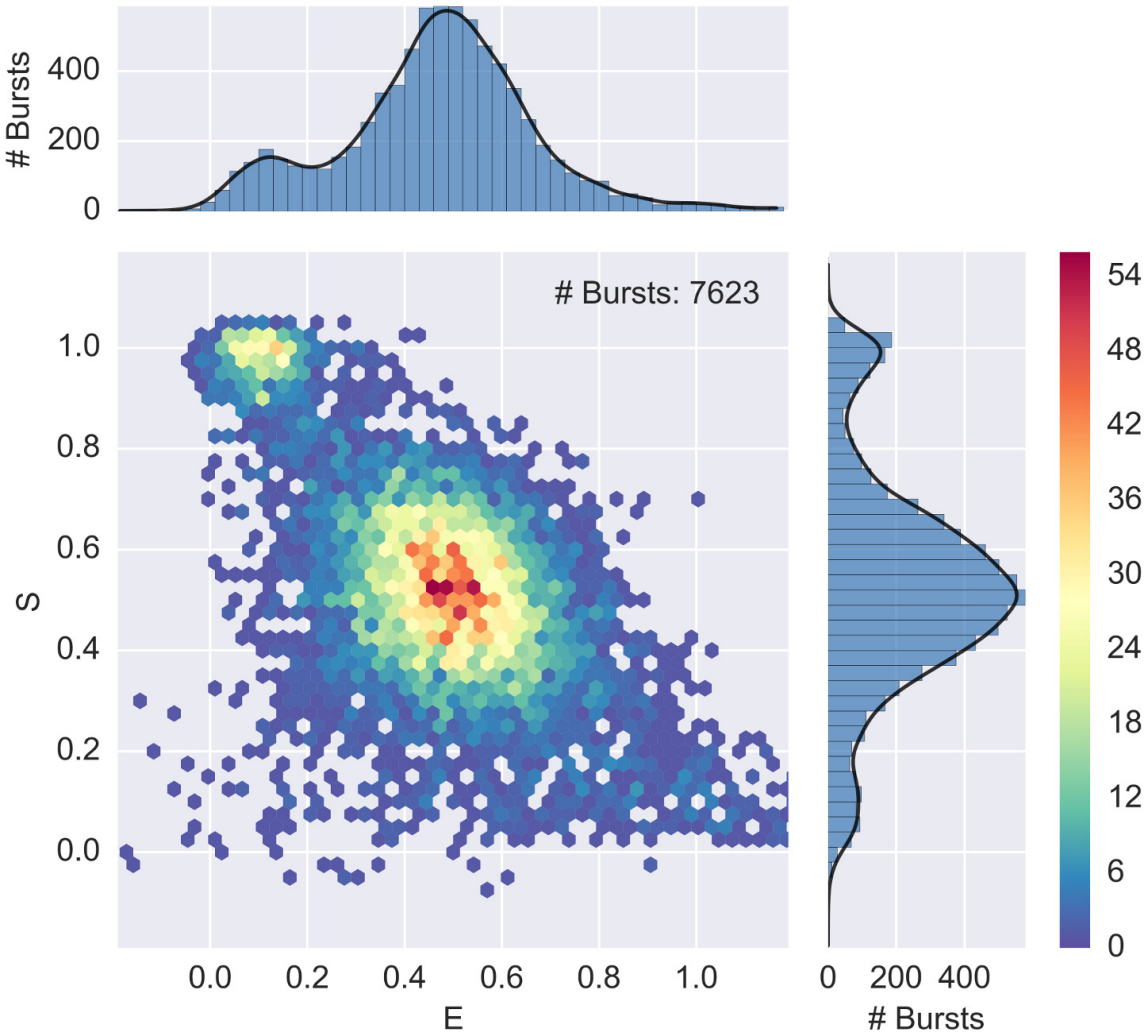
E-S histogram showing FRET, D-only and A-only populations. A 2-D ALEX histogram and marginal E and S histograms for a 40-bp dsDNA with D-A distance of 17 bases (Donor dye: ATTO550, Acceptor dye: ATTO647N). Bursts are selected with a size-threshold of 30 photons, including A_ex_ photons. The plot is obtained with alex_jointplot(ds). The 2D E-S distribution plot (join plot) is an histogram with hexagonal bins, which reduce the binning artifacts (compared to square bins) and naturally resembles a scatter-plot when the burst density is low (see S4 Appendix). Three populations are visible: FRET population (middle), D-only population (top left) and A-only population (bottom, *S* < 0.2). Compare with Fig 5 where the FRET population has been isolated.

The result of this selection is plotted in Fig 4. When add_naa is not specified, as in the previous section, the default is add_naa = False (i.e. compute size using only D_ex_ photons).

Another important parameter for defining the burst size is the *γ*-factor, i.e. the product of the relative quantum yield and detection efficiency of the acceptor versus donor signals. As noted in section Corrected Burst Sizes and Weights, the *γ*-factor is used to compensate for different fluorescence quantum yields for the D and A fluorophores as well as different photon-detection efficiencies for the D and A channels. When *γ* is significantly different from 1, neglecting its effect on burst size leads to over-representing one FRET population versus the others.

When the *γ* factor is known (and ≠1), a more unbiased selection of different FRET populations can be achieved passing the argument gamma to the selection function:

ds = d.select_bursts(select_bursts.size,

       th1 = 15, gamma = 0.65)

When not specified, *γ* = 1 is assumed. For more details on burst size selection, see the select_bursts.size documentation (link).

#### Python details

The method to compute *γ*-corrected burst sizes (with or without addition of naa) is Data.burst_sizes (link).

#### Select the FRET Populations

In smFRET-ALEX experiments, donor-only (D-only) and acceptor-only (A-only) populations can be detected in addition to the FRET population(s). In most cases, the D-only and A-only populations are of no interest and need to be filtered out.

In principle, using the E-S representation, D-only and A-only bursts can be excluded by selecting bursts within a range of *S* values (e.g. S = 0.2-0.8). This approach, however, simply truncates the burst distribution with arbitrary thresholds and is therefore not recommended for quantitative assessment of FRET populations.

An alternative approach consists in applying two selection filters sequentially. First, the A-only population is filtered out by applying a threshold on the number of photons during D excitation (D_ex_). Second, the D-only population is filtered out by applying a threshold on the number of A photons during A excitation (A_ex_A_em_). The commands for these combined selections are:

ds1 = d.select_bursts(select_bursts.size, th1 = 15)

ds2 = ds1.select_bursts(select_bursts.naa, th1 = 15)

**Fig 5:**
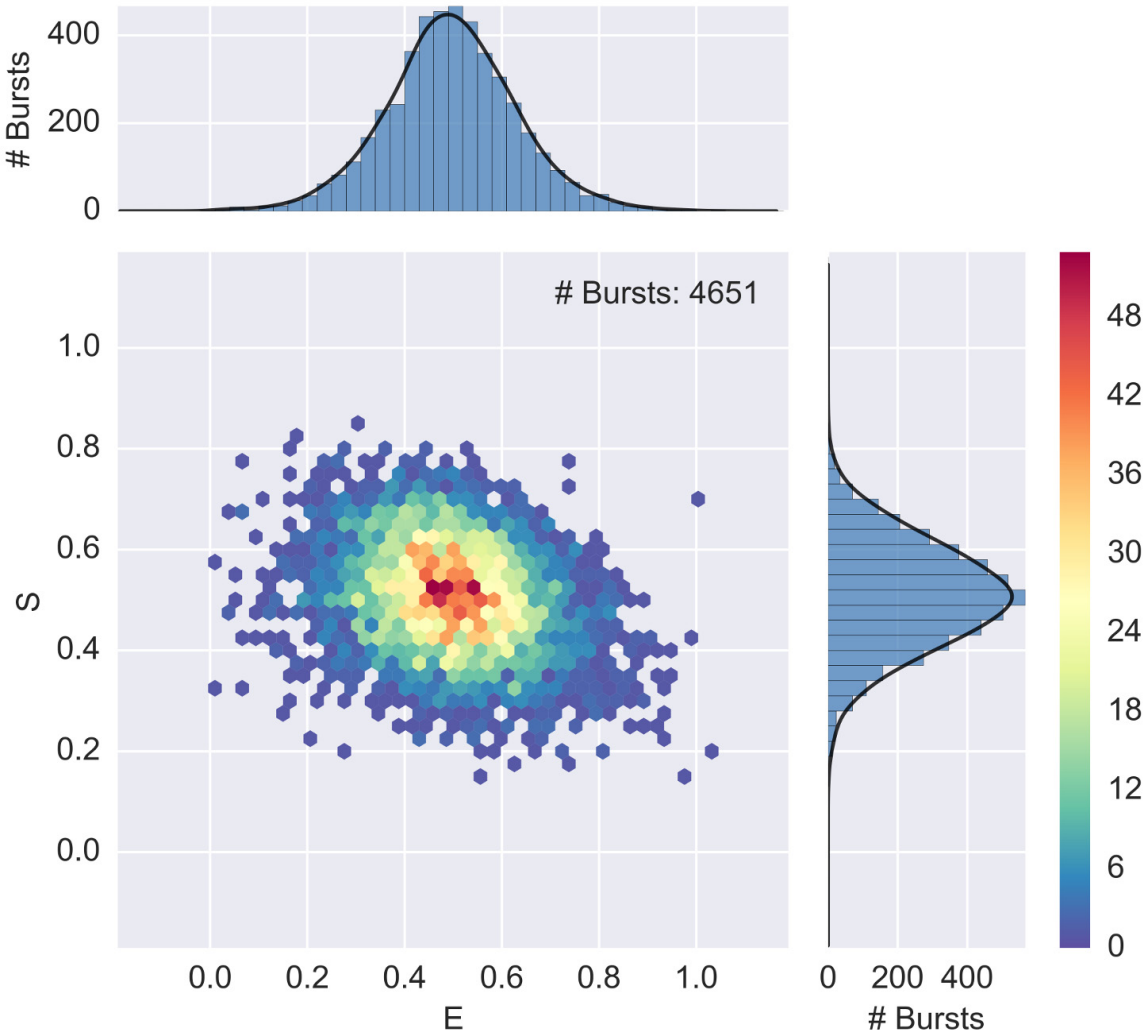
E-S histogram after filtering out D-only and A-only populations. 2-D ALEX histogram after selection of FRET population using the composition of two burst selection filters: (1) selection of bursts with counts in D_ex_ stream larger than 15; (2) selection of bursts with counts in A_ex_A_em_ stream larger than 15. Compare to Fig 4 where all burst populations (FRET, D-only and A-only) are reported.

Here, the variable ds2 contains the combined burst selection. Fig 5 shows the resulting filtered FRET population obtained with the previous selection.

### Population Analysis

Typically, after bursts selection, E or S histograms are fitted to a model. FRETBursts mfit module allows fitting histograms of bursts quantities (i.e. E or S) with arbitrary models. In this context, a model is an object specifying a function, the parameters varied during the fit and optional constraints for these parameters. This concept of model is taken from *lmfit* [51], the underlying library used by FRETBursts to perform the fits.

Models can be created from arbitrary functions. FRETBursts includes predefined (i.e. built-in) models such as 1- to 3-Gaussian peaks or 2-Gaussian connected by a flat “plateau”. The latter is an empirical model which can be used to more accurately fit the center values of two populations when the peaks are connected by intermediate-FRET bursts (for the analytical definition of this function see the documentation, link). Built-in models are created by calling a corresponding factory function (whose names start with mfit.factory_) which initializes the parameters with values and constraints suitable for E and S histograms fits (see *Factory Functions* documentation, link).

As an example, we can fit the E histogram of bursts in the ds variable with two Gaussian peaks with the following command:

bext.bursts_fitter(ds, ‘E’, binwidth = 0.03,

       model = mfit.factory_two_gaussians())

Changing ’E’ with ’S’ will fit the S histogram instead. The binwidth argument specifies the histogram bin width and the model argument defines which model shall be used for fitting.

All fitting results (including best fit values, uncertainties, etc…), are stored in the E_fitter (or S_fitter) attributes of the Data variable (named ds here). To print a comprehensive summary of the fit results, including uncertainties, reduced *χ*^2^ and correlation between parameters, we can use the following command:

fit_res = ds.E_fitter.fit_res[0]

print(fit_res.fit_report())

**Fig 6:**
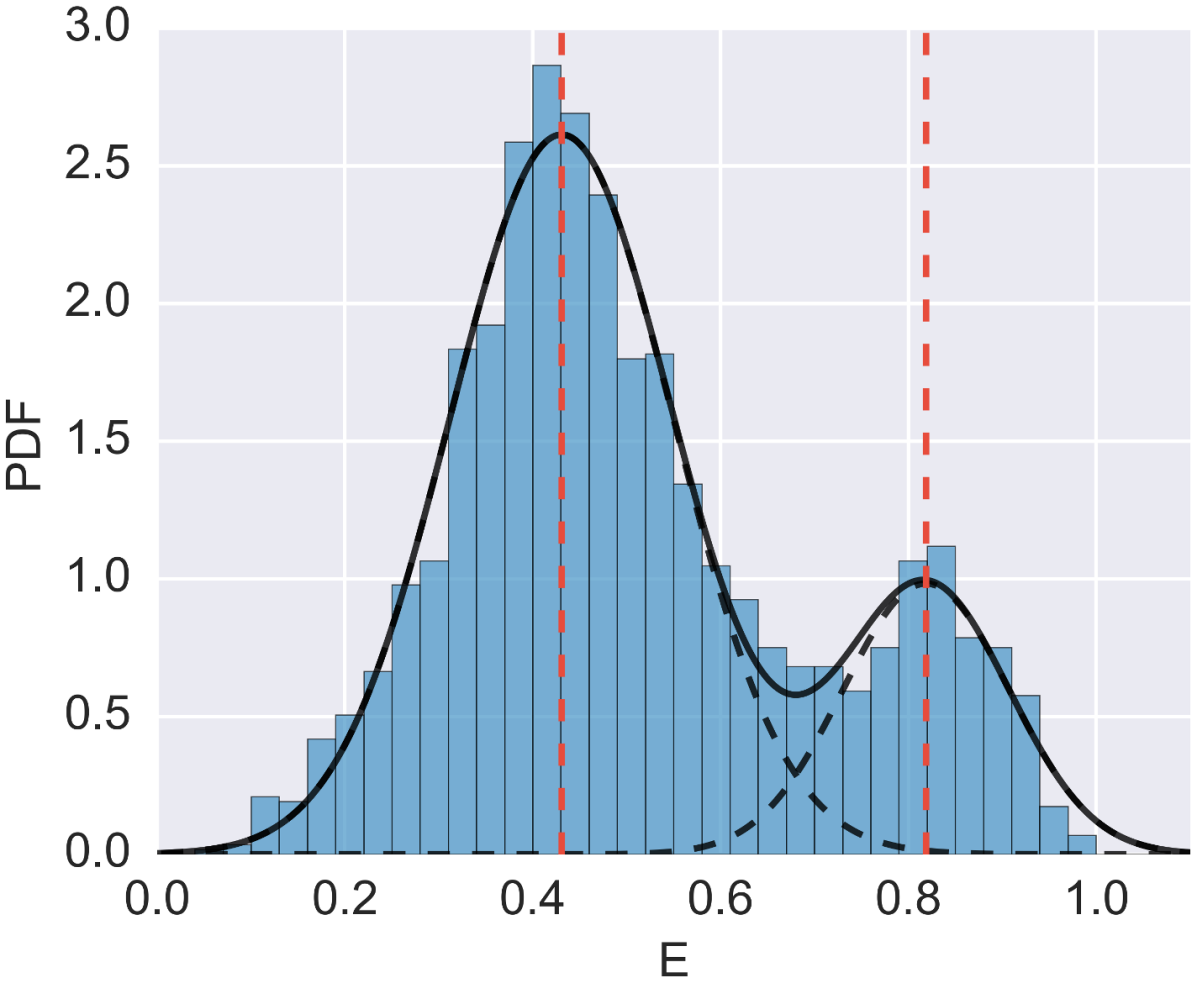
FRET histogram fitted with two Gaussians. Example of a FRET histogram fitted with a 2-Gaussian model. After performing the fit (see main text), the plot is generated with dplot(ds, hist_fret, show_model = True).

Finally, to plot the fitted model together with the FRET histogram, as shown in Fig 6, we pass the parameter show_model = True to the hist_fret function (see S4 Appendix for an introduction to plotting in FRETBursts):

dplot(ds, hist_fret, show_model = True)

For more examples on fitting bursts data and plotting results, refer to the fitting section of the μs-ALEX notebook (link), the *Fitting Framework* section of the documentation (link) as well as the documentation for bursts_fitter function (link).

#### Python details

Models returned by FRETBursts’s factory functions (mfit.factory_*) are lmfit.Model objects (link). Custom models can be created by calling lmfit.Model directly. When an lmfit.Model is fitted, it returns a ModelResults object (link), which contains all information related to the fit (model, data, parameters with best values and uncertainties) and useful methods to operate on fit results. FRETBursts puts a ModelResults object of each excitation spot in the list ds.E_fitter.fit_res. For instance, to obtain the reduced *χ*^2^ value of the E histogram fit in a single-spot measurement d, we use the following command:

d.E_fitter.fit_res[0].redchi

Other useful attributes are aic and bic which contain statistics for the Akaike information criterion (AIC) [52] and the Bayes Information criterion (BIC) [53]. AIC and BIC are general-purpose statistical criteria for comparing the suitability of multiple models according to the data. By penalizing models with higher number of parameters, these criteria strike a balance between the need of achieving high goodness of fit with the need of keeping the model complexity low to avoid overfitting [54].

Examples of definition and modification of fit models are provided in the aforementioned μs-ALEX notebook (link). Users can also refer to the comprehensive lmfit’s documentation (link).

### FRET Dynamics

Single-molecule FRET histograms show more information than just mean FRET efficiencies. While in general the presence of several peaks clearly indicates the existence of multiple subpopulations, a single peak cannot a priori be associated with a single population defined by a unique FRET efficiency without further analysis.

Shot-noise analysis [17] or probability distribution analysis (PDA) [18, 55] allow to compute the minimum width of a static FRET population (i.e. caused by the statistics of discrete photon-detection events). Typically, several mechanisms contribute to the broadening of the experimental FRET peak beyond the shot-noise limit. These include heterogeneities in the sample resulting in a distribution of Förster radii, or actual conformational changes giving rise to a distribution of D-A distances [8].

Gopich and Szabo developed an elegant analytical model for the FRET distribution of *M* interconverting states based on superposition of Gaussian peaks [56]. Unfortunately, the method is not of straightforward application for freely-diffusing data as it requires a special selection criterion for filtering bursts with quasi-Poisson rates. Santoso et al. [57] and Kalinin et al. [58] extended the PDA approach to estimate conversion rates between different states by comparing FRET histograms as a function of the time-bin size. In addition, Gopich and Szabo [59, 60] developed a related method to compute conversion rates using a likelihood function which depends on photon timestamps (overcoming the time binning and FRET histogramming step and directly applicable to freely-diffusing data). In case of measurement including lifetime, the multiparameter fluorescence detection (MFD) method allows to identify dynamics from the deviation from the linear relation between lifetime and E [8]. Hoffman et al. [61] proposed a method called RASP (recurrence analysis of single particles) to extend the timescale of detectable kinetics. Hoffman et al. compute the probability that two nearby bursts are due to the same molecule and therefore allows setting a time-threshold for considering consecutive bursts as the same single-molecule event.

Other interesting approaches include combining smFRET and FCS for detecting and quantifying kinetics on timescales much shorter than the diffusion time [62–64]. In addition, Bayes-based methods have been proposed to fit static populations [65, 66], or to study dynamics [67].

Finally, two related methods for discriminating between static heterogeneity and sub-millisecond dynamics are Burst Variance Analysis (BVA) proposed by Torella et al. [23] and two-channel kernel density estimator (2CDE) proposed by Tomov et al. [24]. The BVA method is described in the next section. The 2CDE method, which has been implemented in FRETBursts, computes local photon rates from timestamps within bursts using Kernel Density Estimation (KDE) (FRETBursts includes general-purpose functions to compute KDE of photon timestamps in the phrates module, (link)). From time variations of local rates, it is possible to infer the presence of some dynamics. In particular, the 2CDE method builds, for each burst, a quantity (*E*)_*D*_ (or (1 − *E*)_*A*_), which is equal to the burst average *E* when no dynamics is present, but is biased toward an higher (or lower) value in presence of dynamics. From these quantities, a burst “estimator” (called FRET-2CDE) is derived. For a user, the 2CDE method amounts to plotting the 2-D histogram of *E* versus FRET-2CDE, and assessing the vertical position of the various populations: populations centered around FRET-2CDE = 10 undergo no dynamics, while population biased towards higher FRET-2CDE values undergo dynamics.

The BVA and 2CDE methods are implemented in two notebooks included with FRETBursts (BVA link, 2CDE link). To use them, a user needs to download the relevant notebook and run the anaysis therein. The other methods mentioned in this section are not currently implemented in FRETBursts. However, users can implement their additional favorite method taking advantage of FRETBursts functions for burst analysis and timestamps/bursts manipulation. To facilitate this task, in the next section, we show how to perform low-level analysis of timestamps and bursts data by implementing the BVA method from scratch. An additional example showing how to split bursts in constant time-bins can be found in the respective FRETBursts notebook (link). These examples serve as a guide for implementing new methods. We welcome researchers willing to implement new methods to ask questions on GitHub or on the mailing list. We also encourage sharing eventual new methods implemented in FRETBursts for the benefit the entire community.

## Implementing Burst Variance Analysis

In this section, we describe how to implement burst variance analysis (BVA) as described in [23]. FRETBursts provides well-tested, general-purpose functions for timestamps and burst data manipulation and therefore simplifies implementing custom burst analysis algorithms such as BVA.

### BVA Overview

The BVA method has been developed to identify the presence of dynamics in FRET distributions [23], and has been successfully applied to identify biomolecular processes with dynamics on the millisecond time-scale [23, 68].

The basic idea behind BVA is to subdivide bursts into contiguous burst chunks (sub-bursts) comprising a fixed number *n* of photons, and to compare the empirical variance of acceptor counts of all sub-bursts in a burst, with the theoretical shot-noise-limited variance. An empirical variance of sub-bursts larger than the shot-noise-limited value indicates the presence of dynamics.

In a FRET (sub-)population originating from a single static FRET efficiency, the sub-bursts acceptor counts *n*_*a*_ can be modeled as a binomial-distributed random variable *N*_*a*_ ∼ B(*n*, *E*_*p*_), where *n* is the number of photons in each sub-burst and *E*_*p*_ is the estimated population proximity-ratio (PR). Note that we can use the PR because, regardless of the molecular FRET efficiency, the detected counts are partitioned between donor and acceptor channels according to a binomial distribution with success probability equal to the PR. The only approximation done here is neglecting the presence of background (a reasonable approximation since the backgrounds counts are in general a very small fraction of the total counts). We refer the interested reader to [23] for further discussion.

If *N*_*a*_ follows a binomial distribution, the random variable *E*_sub_ = *N*_*a*_/*n*, has a standard deviation reported in Eq (3).

$$\begin{array}{c} \text{Std}\left(E_{\text{sub}}\right)={\left(\frac{E_{p}\;\left(1-E_{p}\right)}{n}\right)}^{1/2} \end{array}$$ (3)

**Fig 7:**
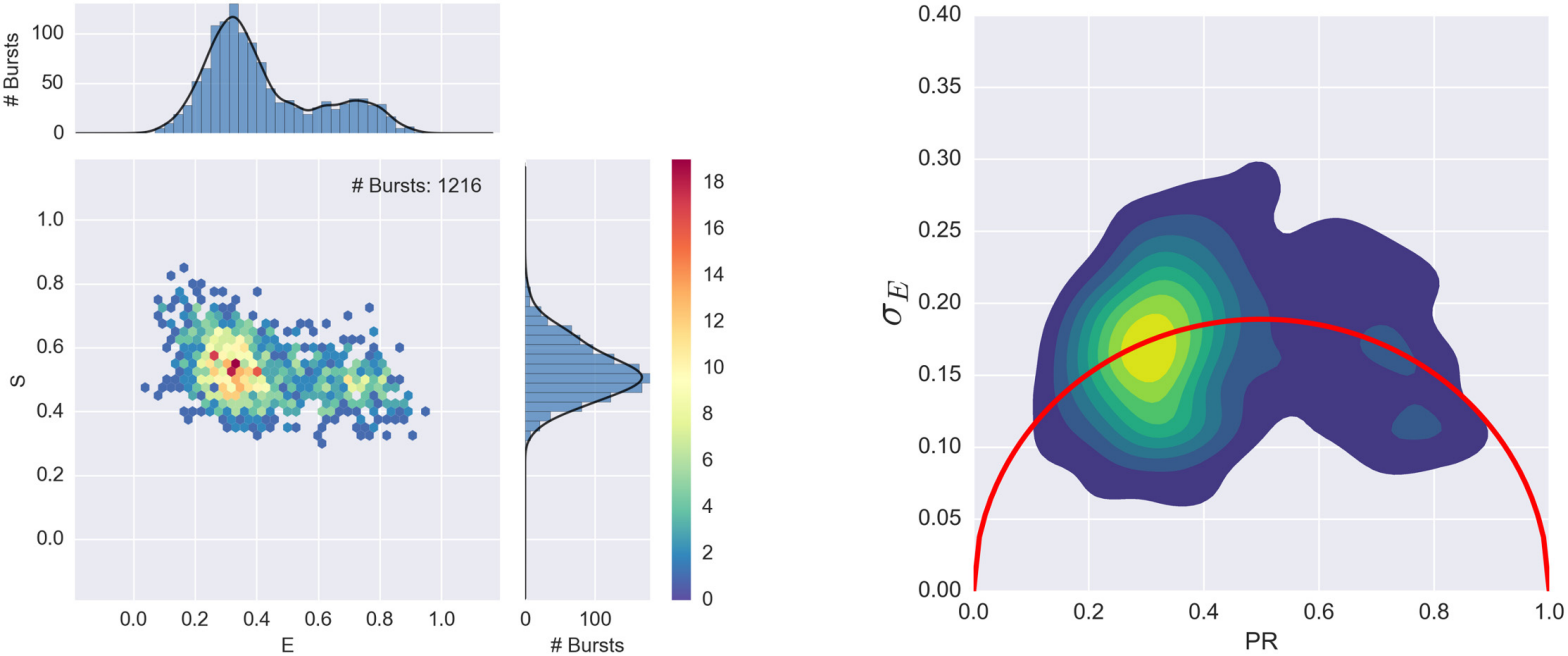
BVA distribution for a static mixture sample. The left panel shows the E-S histogram for a mixture of single stranded DNA (20dT) and double stranded DNA (20dT-20dA) molecules in 200 mM MgCl_2_. The right panel shows the corresponding BVA plot. Since both 20dT and 20dT-20dA are stable and have no dynamics, the BVA plots shows *s*_*E*_ peaks lying on the static standard deviation curve (*red curve*).

**Fig 8:**
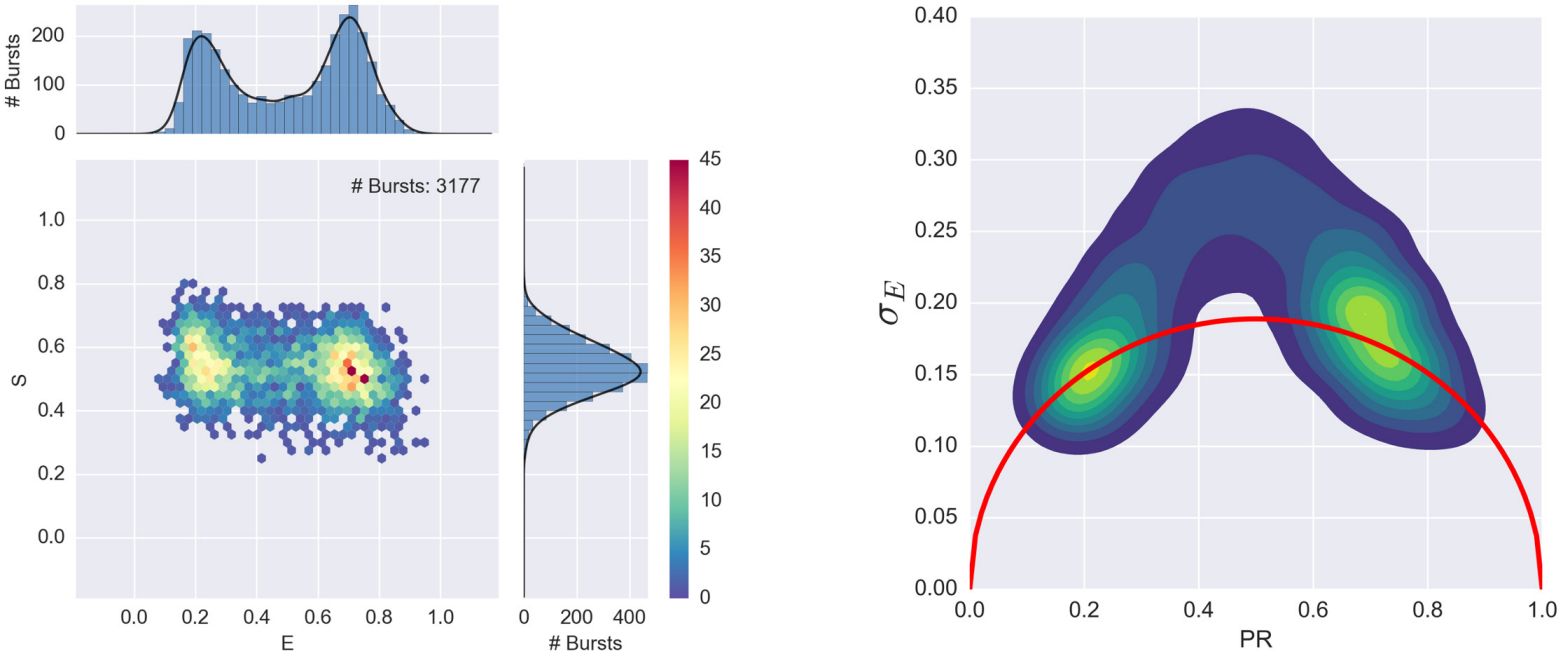
BVA distribution for a hairpin sample undergoing dynamics. The left panel shows the E-S histogram for a single stranded DNA sample (*A*_31_-TA, see text), designed to form a transient hairpin in 400mM NaCl. The right panel shows the corresponding BVA plot. Since the transition between hairpin and open structure causes a significant change in FRET efficiency, *s*_*E*_ lies largely above the static standard deviation curve (*red curve*).

BVA analysis consists of four steps: 1) dividing bursts into consecutive sub-bursts containing a constant number of consecutive photons *n*, 2) computing the PR of each sub-burst, 3) calculating the empirical standard deviation (*s*_*E*_) of sub-bursts PR in each burst, and 4) comparing *s*_*E*_ to the expected standard deviation of a shot-noise-limited distribution (Eq (3)). If, as in Fig 7, the observed FRET efficiency distribution originates from a static mixture of sub-populations (of different non-interconverting molecules) characterized by distinct FRET efficiencies, *s*_*E*_ of each burst is only affected by shot-noise and will follow the expected standard deviation curve based on Eq (3). Conversely, if the observed distribution originates from biomolecules belonging to a single species, which interconverts between different FRET sub-populations (over times comparable to the diffusion time), as in Fig 8, *s*_*E*_ of each burst will be larger than the expected shot-noise-limited standard deviation, and will be located above the shot-noise standard deviation curve (right panel of Fig 8). Sample in Fig 8 is single stranded DNA (*A*_31_-TA, as described in [69]), designed to form a transient hairpin in 400mM NaCl.

### BVA Implementation

The following paragraphs describe the low-level details involved in implementing the BVA using FRETBursts. The main goal is to illustrate a real-world example of accessing and manipulating timestamps and burst data. For a ready-to-use BVA implementation users can refer to the corresponding notebook included with FRETBursts (link).

#### Python details

For BVA implementation, two photon streams are needed: all-photons during donor excitation (D_ex_) and acceptor photons during donor excitation (D_ex_A_em_). These photon stream selections are obtained by computing boolean masks as follows (see S3 Appendix):

Dex_mask = ds.get_ph_mask(ph_sel = Ph_sel(Dex=’DAem’))

DexAem_mask = ds.get_ph_mask(ph_sel = Ph_sel(Dex=’Aem’))

DexAem_mask_d = AemDex_mask[Dex_mask]

Here, the first two variables (Dex_mask and DexAem_mask) select photon from the all-photons timestamps array, while DexAem_mask_d, selects A-emitted photons from the array of photons emitted during D-excitation. As shown below, the latter is needed to count acceptor photons in burst chunks.

Next, we need to express bursts start-stop data as indexes of the D-excitation photon stream (by default burst start-stop indexes refer to all-photons timestamps array):

ph_d = ds_FRET.get_ph_times(ph_sel = Ph_sel(Dex=’DAem’))

bursts = ds_FRET.mburst[0]

bursts_d = bursts.recompute_index_reduce(ph_d)

Here, ph_d contains the D_ex_ timestamps, bursts the original burst data and bursts_d the burst data with start-stop indexes relative to ph_d.

Finally, with the previous variables at hand, the BVA algorithm can be easily implemented by computing the *s*_*E*_ quantity for each burst:

n = 7

E_sub_std = []

for burst in bursts_d:

  E_sub = []

  startlist = range(burst.istart, burst.istop + 2—n, n)

  stoplist = [i + n for i in startlist]

  for start, stop in zip(startlist, stoplist):

   A_D = DexAem_mask_d[start:stop].sum()

   E = A_D / n

   E_sub.append(E)

  E_sub_std.append(np.std(E_sub))

Here, n is the BVA parameter defining the number of photons in each burst chunk. The outer loop iterates through bursts, while the inner loop iterates through sub-bursts. The variables startlist and stoplist are the list of start-stop indexes for all sub-bursts in current burst. In the inner loop, A_D and E contain the number of acceptor photons and FRET efficiency for the current sub-burst. Finally, for each burst, the standard deviation of E is appended to the list E_sub_std.

By plotting the 2D distribution of *s*_*E*_ (i.e. E_sub_std) versus the average (uncorrected) E we obtain the BVA plots of Figs 7 and 8.

## Conclusions

FRETBursts is an open source and openly developed (see S2 Appendix) implementation of established smFRET burst analysis methods made available to the single-molecule community. It implements several novel concepts which improve the analysis results, such as time-dependent background estimation, background-dependent burst search threshold, burst weighting and *γ*-corrected burst size selection. More importantly, FRETBursts provides a library of thoroughly-tested functions for timestamps and burst manipulation, making it an ideal platform for developing and comparing new analytical techniques.

We envision FRETBursts both as a state-of-the-art burst analysis software as well as a platform for development and assessment of novel algorithms. To underpin this envisioned role, FRETBursts is developed following modern software engineering practices, such as DRY principle (link) to reduce duplication and KISS principle (link) to reduce over-engineering. Furthermore, to minimize the number software errors [36, 70], we employ defensive programming [39] which includes code readability, unit and regression testing and continuous integration [28]. Finally, being open source, any scientist can inspect the source code, fix errors, adapt it to her own needs.

We believe that, in the single-molecule community, standard open source software implementations, such as FRETBursts, can enhance reliability and reproducibility of analysis and promote a faster adoption of novel methods, while reducing the duplication of efforts among different groups.

## Supporting Information

S1 Appendix Notebook Workflow. A description of the notebook workflow used by FRETBursts.
(PDF)

S2 Appendix Development and Contributions. A description of development philosophy and techniques as well as how to contribute to the FRETBursts project.
(PDF)

S3 Appendix Timestamps and Burst Data. General concepts of how timestamps and bursts data are stored and handled in FRETBursts.
(PDF)

S4 Appendix Plotting Data. A description of the syntax used to perform plots in FRETBursts and of the 2-D hexagonal-bin histogram used in E-S plots.
(PDF)

S5 Appendix Background Estimation With Optimal Threshold. A description of the algorithm used by FRETBursts to compute the optimal threshold for background estimation.
(PDF)

S6 Appendix Burst Weights. Theory underpinning the choice of using burst size as weights for FRET estimation.
(PDF)
