# Supplementary material for: FRETBursts: An Open Source Toolkit for Analysis of Freely-Diffusing Single-Molecule FRET
Source: PLoS One. 2016 Aug 17;11(8):e0160716. doi: 10.1371/journal.pone.0160716 (PMC4988647; doi:10.1371/journal.pone.0160716)
Supplement: S1 Appendix — A description of the notebook workflow used by FRETBursts. (PDF) [file pone.0160716.s001.pdf]

Support Information

FRETBursts: An Open Source Toolkit for Analysis of Freely-Diffusing Single-Molecule FRET

Antonino Ingargiola*[1], Eitan Lerner[1], SangYoon Chung[1], Shimon Weiss[1], and Xavier Michalet[1]

[1]Dept. Chem. & Biochem, Univ. California Los Angeles, Los Angeles, CA, USA.

S1 Appendix. Notebook Workflow

FRETBursts has been developed with the goal of facilitating computational reproducibility of the performed data analysis [1]. For this reason, the preferential way of using FRETBursts is by executing one of the tutorials which are in the form of Jupyter notebooks [2]. Jupyter (formerly IPython) notebooks are web-based documents which contain both code and rich text (including equations, hyperlinks, figures, etc...). FRETBursts tutorials are notebooks which can be re-executed, modified or used to process new data files with minimal modifications. The "notebook workflow" [2] not only facilitates the description of the analysis (by integrating the code in a rich document) but also greatly enhances its reproducibility by storing an execution trail that includes software versions, input files, parameters, commands and all the analysis results (text, figures, tables, etc.).

The Jupyter Notebook environment streamlines FRETBursts execution (compared to a traditional script and terminal based approach) and allows FRETBursts to be used even without prior python knowledge. The user only needs to get familiar with the notebook graphical environment, in order to be able to navigate and run the notebooks. A list of all FRETBursts notebooks can be found in the `FRETBursts_notebooks` repository on GitHub (link). Finally, we provide a service to run FRETBursts notebooks online, without requiring any software installation (link).

References

[1] Jonathan B. Buckheit and David L. Donoho. WaveLab and Reproducible Research. In *Wavelets and Statistics*, volume 103, pages 55–81. Springer Science + Business Media, 1995. doi:10.1007/978-1-4612-2544-7_5.

[2] Helen Shen. Interactive notebooks: Sharing the code. *Nature*, 515(7525):151–152, nov 2014. doi:10.1038/515151a.

*ingargiola.antonino@gmail.com
