# Supplementary material for: FRETBursts: An Open Source Toolkit for Analysis of Freely-Diffusing Single-Molecule FRET
Source: PLoS One. 2016 Aug 17;11(8):e0160716. doi: 10.1371/journal.pone.0160716 (PMC4988647; doi:10.1371/journal.pone.0160716)
Supplement: S2 Appendix — A description of development philosophy and techniques as well as how to contribute to the FRETBursts project. (PDF) [file pone.0160716.s002.pdf]

Support Information

FRETBursts: An Open Source Toolkit for Analysis of Freely-Diffusing Single-Molecule FRET

Antonino Ingargiola*[1], Eitan Lerner[1], SangYoon Chung[1], Shimon Weiss[1], and Xavier Michalet[1]

[1]Dept. Chem. & Biochem, Univ. California Los Angeles, Los Angeles, CA, USA.

S2 Appendix. Development and Contributions

Errors are an inevitable reality in any reasonably complex software [1, 2]. It is therefore critical to implement counter-measures to minimize the probability of introducing bugs and their potential impact [3, 4]. In developing FRETBursts we leverage open source technlogies and follow modern software development best-practices as summarized below.

FRETBursts (and the entire python ecosystem it depends on) is open source and the source code is fully available for any scientist to study, review and modify. The open source nature of FRETBursts and of the python ecosystem, not only makes it a more transparent, reviewable platform for scientific data analysis, but also allows to leverage state-of-the-art online services such as GitHub (link) for hosting, issues tracking and code reviews, TravisCI (link) and AppVeyor (link) for continuous integration (i.e. automated test suite execution on multiple platforms after each commit) and ReadTheDocs.org for automatic documentation building and hosting. All these services would be extremely costly, if available at all, for a proprietary software or platform [5].

We highly value source code readability, a property which can reduce the number of bugs by facilitating understanding and verifying the code. For this purpose, FRETBursts code-base is well commented (with comments representing over 35% of the source code), follows the PEP8 python code style rules (link), and has docstrings in napoleon format (link).

Reference documentation is built with Sphinx (sphinx-doc.org) and all API documents are automatically generated from docstrings. On each commit, documentation is automatically built and deployed on ReadTheDocs.org.

Unit tests cover most of the core algorithms, ensuring consistency and minimizing the probability of introducing bugs. The continuous integration services, execute the full test suite on each commit on multiple platforms, immediately reporting errors. As a rule, whenever a bug is discovered, the fix also includes a new test to ensure that the same bug does not happen in the future. In addition to the unit tests, we include a regression-test notebook (link) to easily compares numerical results between two versions of FRETBursts. Additionally, the tutorials themselves are executed before each release as an additional test layer to ensure that no errors or regressions are introduced.

FRETBursts is openly developed using the GitHub platform. The authors encourage users to use GitHub issues for questions, discussions and bug reports, and to submit patches through GitHub pull requests. Contributors of any level of expertise are welcome in the projects and publicly acknowledged. Contributions can be as simple as pointing out deficiencies in the documentation but can also be bug reports or corrections to the documentation or code. Users willing to implement new features are encouraged to open an Issue on GitHub and to submit a Pull Request. The open source nature of FRETBursts guarantees that contributions will become available to the entire single-molecule community.

References

[1] Zeeya Merali. Computational science: ...Error. *Nature*, 467(7317):775–777, oct 2010. doi:10.1038/467775a.

[2] David A. W. Soergel. Rampant software errors may undermine scientific results. *F1000Research*, jul 2015. doi:10.12688/f1000research.5930.2.

*ingargiola.antonino@gmail.com

[3] Andreas Prlić and James B. Procter. Ten Simple Rules for the Open Development of Scientific Software. *PLoS Computational Biology*, 8(12):e1002802, dec 2012. `doi:10.1371/journal.pcbi.1002802`.

[4] Greg Wilson, D. A. Aruliah, C. Titus Brown, Neil P. Chue Hong, Matt Davis, Richard T. Guy, Steven H. D. Haddock, Kathryn D. Huff, Ian M. Mitchell, Mark D. Plumbley, Ben Waugh, Ethan P. White, and Paul Wilson. Best Practices for Scientific Computing. *PLoS Biology*, 12(1):e1001745, jan 2014. `doi:10.1371/journal.pbio.1001745`.

[5] Jeremy Freeman. Open source tools for large-scale neuroscience. *Current Opinion in Neurobiology*, 32:156–163, jun 2015. `doi:10.1016/j.conb.2015.04.002`.
