# Supplementary material for: FRETBursts: An Open Source Toolkit for Analysis of Freely-Diffusing Single-Molecule FRET
Source: PLoS One. 2016 Aug 17;11(8):e0160716. doi: 10.1371/journal.pone.0160716 (PMC4988647; doi:10.1371/journal.pone.0160716)
Supplement: S3 Appendix — General concepts of how timestamps and bursts data are stored and handled in FRETBursts. (PDF) [file pone.0160716.s003.pdf]

Support Information

FRETBursts: An Open Source Toolkit for Analysis of Freely-Diffusing Single-Molecule FRET

Antonino Ingargiola*[1], Eitan Lerner[1], SangYoon Chung[1], Shimon Weiss[1], and Xavier Michalet[1]

[1]Dept. Chem. & Biochem, Univ. California Los Angeles, Los Angeles, CA, USA.

S3 Appendix. Timestamps and Burst Data

Beyond providing prepackaged functions for established methods, FRETBursts also provides the infrastructure for exploring new analysis approaches. Users can easily get timestamps (or selection masks) for any photon stream. Core burst data (start and stop times, indexes and derived quantities for each burst) are stored in `Bursts` objects (link). This object provides a simple and well-tested interface (100 % unit-test coverage) to access and manipulate burst data. `Bursts` are created from a sequence of start/stop times and indexes, while all other fields are automatically computed. `Bursts`'s methods allow to recompute indexes relative to a different photon selection or recompute start and stop times relative to a new timestamps array. Additional methods perform fusion of nearby bursts or combination of two set of bursts (time intersection or union). This functionality is used for example to implement the DCBS. In conclusion, `Bursts` efficiently implements all the common operations performed with burst data, providing and easy-to-use interface and well tested algorithms. Leveraging `Bursts` methods, users can implement new types of analysis without wasting time implementing (and debugging) standard manipulation routines. Examples of working directly with timestamps, masks (i.e. photon selections) and burst data are provided in one of the FRETBursts notebooks (link). Section *Implementing Burst Variance Analysis* provides a complete example on using FRETBursts to implement custom burst analysis techniques.

Python details Timestamps are stored in the `Data` attribute `ph_times_m`, which is a list or arrays, one array per excitation spot. In single-spot measurements the full timestamps array is accessed as `Data.ph_times_m[0]`. To get timestamps of arbitrary photon streams, users can call `Data.get_ph_times` (link). Photon streams are selected from the full (all-photons) timestamps array using boolean masks, which can be obtained calling `Data.get_ph_mask` (link). All burst data (e.g. start-stop times and indexes, burst duration, etc.) are stored in `Bursts` objects. For uniformity, the bursts start-stop indexes are always referring to the all-photons timestamps array, regardless of the photon stream used for burst search. `Bursts` objects internally store only start and stop times and indexes. The other `Bursts` attributes (duration, photon counts, etc.) are computed on-the-fly when requested (using class properties), thus minimizing the object state. `Bursts` support iteration with performances similar to iterating through rows of 2D row-major numpy arrays.

*ingargiola.antonino@gmail.com
