# Supplementary material for: FRETBursts: An Open Source Toolkit for Analysis of Freely-Diffusing Single-Molecule FRET
Source: PLoS One. 2016 Aug 17;11(8):e0160716. doi: 10.1371/journal.pone.0160716 (PMC4988647; doi:10.1371/journal.pone.0160716)
Supplement: S4 Appendix — A description of the syntax used to perform plots in FRETBursts and of the 2-D hexagonal-bin histogram used in E-S plots. (PDF) [file pone.0160716.s004.pdf]

Support Information

FRETBursts: An Open Source Toolkit for Analysis of Freely-Diffusing Single-Molecule FRET

Antonino Ingargiola*[1], Eitan Lerner[1], SangYoon Chung[1], Shimon Weiss[1], and Xavier Michalet[1]

[1]Dept. Chem. & Biochem, Univ. California Los Angeles, Los Angeles, CA, USA.

S4 Appendix. Plotting `Data`

FRETBursts uses matplotlib [1] and seaborn [2] to provide a wide range of built-in plot functions (link) for `Data` objects. The plot syntax is the same for both single and multi-spot measurements. The majority of plot commands are called through the wrapper function `dplot`, for example to plot a timetrace of the photon data, type:

```
dplot(d, timetrace)
```

The function `dplot` is the generic plot function, which creates figure and handles details common to all the plotting functions (for instance, the title). `d` is the `Data` variable and `timetrace` is the actual plot function, which operates on a single channel. In multispot measurements, `dplot` creates one subplot for each spot and calls `timetrace` for each channel.

All built-in plot functions which can be passed to `dplot` are defined in the `burst_plot` module (link).

Python details When FRETBursts is imported, all plot functions are also imported. To facilitate finding the plot functions through auto-completion, their names start with a standard prefix indicating the plot type. The prefixes are: `timetrace` for binned timetraces of photon data, `ratetrace` for rates of photons as a function of time (non binnned), `hist` for functions plotting histograms and `scatter` for scatter plots. Additional plots can be easily created directly with matplotlib.

By default, in order to speed-up batch processing, FRETBursts notebooks display plots as static images using the *inline* matplotlib backend. User can switch to interactive figures inside the browser by activating the interactive backend with the command `%matplotlib notebook`. Another option is displaying figures in a new standalone window using a desktop graphical library such as QT4. In this case, the command to be used is `%matplotlib qt`.

A few plot functions, such as `timetrace` and `hist2d_alex`, have interactive features which require the QT4 backend. As an example, after switching to the QT4 backend the following command:

```
dplot(d, timetrace, scroll=True, bursts=True)
```

will open a new window with a timetrace plot with overlay of bursts, and an horizontal scroll-bar for quick "scrolling" throughout time. The user can click on a burst to have the corresponding burst info be printed in the notebook. Similarly, calling the `hist2d_alex` function with the QT4 backend allows selecting an area on the E-S histogram using the mouse.

```
dplot(ds, hist2d_alex, gui_sel=True)
```

The values which identify the region are printed in the notebook and can be passed to the function `select_bursts.ES` to select bursts inside that region (see section *Burst Selection* in the main text).

Plotting ALEX histograms

E-S histograms are traditionally computed using a bin size of 0.02-0.04, and cover a range slightly larger than the [0, 1] interval in which ratios of quantities not corrected for background would normally fall. FRETBursts allows plotting the square-bin 2-D E-S histogram using the plot function `hist2d_alex` as shown in the previous example. The histogram can be "smothed" via bicubic interpolation between bin centers (default) or plotted with raw square bins (pass the argument

*ingargiola.antonino@gmail.com

`interpolation='none'`). Additionally, a scatter plot of E-S points is by default overlayed and it can be useful for observing the burst distribution in sparse regions. However, the different layers make this plot hard to read.

A more elegant approach for effectively representing E-S histograms with minimal clutter and high information content is using an hexbin plot (as used by the FRETBursts function `alex_jointplot`). The hexbin plot is a 2-D histograms using hexagonal bins that reduces gridding artifacts of square bins. In addition, in sparse regions, the hexbin plot naturally resembles a scatter plot (with hexagonal markers). The use of hexagonal bins for 2D distributions has been pioneered by Dan Carr in S-PLUS and then popularized by Nicholas Lewin-Koh which wrote the R language port. Later, hexbin has been implemented in the matplotlib python library (which is what FRETBursts uses). The advantages of hexagonal bins have been extensively studied and can be summarized with Nicholas Lewin-Koh words (link):

> Why hexagons? There are many reasons for using hexagons, at least over squares. Hexagons have symmetry of nearest neighbors which is lacking in square bins. Hexagons are the maximum number of sides a polygon can have for a regular tesselation of the plane, so in terms of packing a hexagon is 13% more efficient for covering the plane than squares. This property translates into better sampling efficiency at least for elliptical shapes. Lastly hexagons are visually less biased for displaying densities than other regular tesselations.

The function `alex_jointplot` plots a 3-panels plots with a central hexbin plot of E-S values and marginal E and S histograms represented in top and right panel (see figure 4 and 5 in the main text). Note that unlike other plot functions in FRETBursts, `alex_jointplot` is called directly and not through the `dplot` wrapper.

References

[1] Michael Droettboom, John Hunter, Thomas A Caswell, Eric Firing, Jens Hedegaard Nielsen, Phil Elson, Benjamin Root, Darren Dale, Jae-Joon Lee, Jouni K. Seppänen, Damon McDougall, Andrew Straw, Ryan May, Nelle Varoquaux, Tony S Yu, Eric Ma, Charlie Moad, Steven Silvester, Christoph Gohlke, Peter Würtz, Thomas Hisch, Federico Ariza, Cimarron, Ian Thomas, James Evans, Paul Ivanov, Jeff Whitaker, Paul Hobson, mdehoon, and Matt Giuca. matplotlib: matplotlib v1.5.1, jan 2016. doi:10.5281/zenodo.44579.

[2] Michael Waskom, Olga Botvinnik, drewokane, Paul Hobson, Yaroslav Halchenko, Saulius Lukauskas, Jordi Warmenhoven, John B. Cole, Stephan Hoyer, Jake Vanderplas, gkunter, Santi Villalba, Eric Quintero, Marcel Martin, Alistair Miles, Kyle Meyer, Tom Augspurger, Tal Yarkoni, Pete Bachant, Constantine Evans, Clark Fitzgerald, Tamas Nagy, Erik Ziegler, Tobias Megies, Daniel Wehner, Samuel St-Jean, Luis Pedro Coelho, Gregory Hitz, Antony Lee, and Luc Rocher. seaborn: v0.7.0, January 2016. doi:10.5281/zenodo.45133.
