# Supplementary material for: FRETBursts: An Open Source Toolkit for Analysis of Freely-Diffusing Single-Molecule FRET
Source: PLoS One. 2016 Aug 17;11(8):e0160716. doi: 10.1371/journal.pone.0160716 (PMC4988647; doi:10.1371/journal.pone.0160716)
Supplement: S5 Appendix — A description of the algorithm used by FRETBursts to compute the optimal threshold for background estimation. (PDF) [file pone.0160716.s005.pdf]

Support Information

FRETBursts: An Open Source Toolkit for Analysis of Freely-Diffusing Single-Molecule FRET

Antonino Ingargiola*[1], Eitan Lerner[1], SangYoon Chung[1], Shimon Weiss[1], and Xavier Michalet[1]

[1]Dept. Chem. & Biochem, Univ. California Los Angeles, Los Angeles, CA, USA.

S5 Appendix. Background Estimation With Optimal Threshold

The functions used to fit the background (i.e. `bg.exp_fit` and other functions in `bg` module) provide also a goodness-of-fit estimator computed from the empirical distribution function (EDF) [1, 2]. The "distance" between the EDF and the theoretical (i.e. exponential) cumulative distribution represents and indicator of the quality of fit. Two different distance metrics can be returned by the background fitting functions. The first is the Kolgomorov-Smirnov statistics, which uses the maximum of the difference between the EDF and the theoretical distribution. The second is the Cramér von Mises statistics corresponding to the integral of the squared residuals (see the code for more details, link).

In principle, the optimal inter-photon delay threshold will minimize the error metric. This approach is implemented by the function `calc_bg_brute` (link) which performs a brute-force search in order to find the optimal threshold. This optimization is not necessary under typical experimental conditions, because the estimated rates normally change only a by a few per-cent compared to the heuristic threshold selection used by default.

References

[1] M. A. Stephens. EDF Statistics for Goodness of Fit and Some Comparisons. *Journal of the American Statistical Association*, 69(347):730, sep 1974. doi:10.2307/2286009.

[2] William C. Parr and William R. Schucany. Minimum Distance and Robust Estimation. *Journal of the American Statistical Association*, 75(371):616, sep 1980. doi:10.2307/2287658.

*ingargiola.antonino@gmail.com
