# Supplementary material for: FRETBursts: An Open Source Toolkit for Analysis of Freely-Diffusing Single-Molecule FRET
Source: PLoS One. 2016 Aug 17;11(8):e0160716. doi: 10.1371/journal.pone.0160716 (PMC4988647; doi:10.1371/journal.pone.0160716)
Supplement: S6 Appendix — Theory underpinning the choice of using burst size as weights for FRET estimation. (PDF) [file pone.0160716.s006.pdf]

Support Information

FRETBursts: An Open Source Toolkit for Analysis of Freely-Diffusing Single-Molecule FRET

Antonino Ingargiola*[1], Eitan Lerner[1], SangYoon Chung[1], Shimon Weiss[1], and Xavier Michalet[1]

[1]Dept. Chem. & Biochem, Univ. California Los Angeles, Los Angeles, CA, USA.

S6 Appendix. Burst Weights

S6.1 Theory

Freely-diffusing molecules across a Gaussian excitation volume give rise to a burst size distribution that is exponentially distributed. In a static FRET population, burst counts in the acceptor channel can be modeled as a binomial random variable (RV) with success probability equal to the population PR and number of trials equal to the burst size $n_d + n_a$. Similarly, the PR of each burst E_i (i being the burst index) is simply a binomial divided by the number of trials, with variance reported in eq. 1.

$$\mathrm{Var}(E_i) = \frac{E_p\,(1 - E_p)}{n_{ti}} \tag{1}$$

Bursts with higher counts, provide more accurate estimations of the population PR, since their PR variance is smaller (eq. 1). Therefore, in estimating the population PR we need to "focus" on bigger bursts. Traditionally, this is accomplished by merely discarding bursts below a size-threshold. In the following paragraphs we demonstrate how, by proper weighting bursts, is possible to obtains optimal estimates of PR which takes into account the information of the entire burst population.

According to the Cramer-Rao lower bound (eq. 2), the Fisher information $\mathcal{I}(\theta)$ sets a lower bound on the variance of any statistics $\hat{p}$ of a RV θ.

$$\mathrm{Var}\left(\hat{p}\right) \geq \frac{1}{\mathcal{I}(\theta)} \tag{2}$$

When the statistics $\hat{p}$ is an unbiased estimator of a distribution parameter and the equality holds in eq. 2, the estimator is a minimum-variance unbiased (MVUB) estimator and it is said to be efficient (meaning that it does an optimal use the information contained in the sample to estimate the parameter).

A population of N bursts can be modeled by a set of N binomial variables with same success probability E_p and varying number of successes equal to the burst size. An estimator for E_p can be constructed noticing that the sum of binomial RV with same success probability is still a binomial (with number of trials equal to the sum of the number of trials). Taking the sum of acceptor counts over all bursts divided by the total number of photons as in eq. 3, we obtain an estimator $\hat{E}$ of the proportion of successes.

$$\hat{E} = \frac{\sum_i n_{ai}}{\sum_i n_{ti}} \tag{3}$$

The variance of $\hat{E}$ (eq. 4) is equal to the inverse of the Fisher information $\mathcal{I}(\hat{E})$ and therefore $\hat{E}$ is a MVUB estimator for E_p.

$$\mathrm{Var}(\hat{E}) = \frac{E_p(1 - E_p)}{\sum_i n_{ti}} = \frac{1}{\mathcal{I}(\hat{E})} \tag{4}$$

*ingargiola.antonino@gmail.com

We can finally verify that $\hat{E}$ is equal to the weighted average of the bursts PR E_i (eq. 6), with weights proportional to the burst size (eq. 5).

$$w_i = \frac{n_{ti}}{\sum_i n_{ti}} \tag{5}$$

$$\hat{E}_w = \frac{1}{N}\sum_i w_i E_i = \frac{1}{N}\frac{\sum_i n_{ti}\frac{n_{ai}}{n_{ti}}}{\sum_i n_{ti}} = \hat{E} \tag{6}$$

Since $\hat{E}$ is the MVUB estimator, any other estimator of E_p (in particular the unweighted mean of E_i) will have a larger variance.

We can extend these consideration of optimal weights for the PR estimator to the FRET distribution plot (histograms or KDEs). Building an unweighted histogram (and fitting the peak) is analogous to estimating the E_p with an unweighted average. Conversely, building the FRET histogram using the burst size as weights is equivalent to using the MVUB estimator for E_p.

S6.2 Weighted FRET estimator

Here we report a simple verification of the results of previous section, namely that a weighted mean of E_i is the estimator with minimal variance of E_p. For this purpose, we generated a static FRET population of 100 bursts by simply extracting burst-sizes from an exponential distribution ($\lambda = 10$) and acceptor counts from a binomial distribution ($E_p = 0.2$). By repeatedly fitting the population parameter E_p using a size-weighted and unweighted average, we verified that the former has systematically lower variance of the latter as predicted by the theory (in the current example the unweighted estimator has $28.6\,\%$ higher variance). Note that this result holds for any arbitrary distribution of burst sizes. The full simulation including exponential and gamma-distributed burst sizes is reported in the accompanying Jupyter notebook (link).

S6.3 Weighted FRET histogram

The effect of weighting the FRET histogram is here illustrated with a simulation of a mixture of two static FRET populations and then with experimental data.

We performed a realistic simulation of a static mixture of two FRET populations starting from 3-D Brownian motion diffusion of N particles excited by a numerically computed (non-Gaussian) PSF. Input parameters of the simulation include diffusion coefficient, particle brightness, the two FRET efficiencies, as well as detectors DCR. The simulation is performed with the open source software PyBroMo [1] which creates smFRET data files (i.e. timestamps and detectors arrays) in Photon-HDF5 format [2]. The simulated data file is processed with FRETBursts performing burst search, and only a minimal burst size selection of with threshold of 10 photons. The resulting weighted and unweighted FRET histograms are reported in figure S1. We notice that the use of the weights results in better definition of FRET peaks.

As a final comparison, we report the weighted and unweighted FRET histogram of an experimental FRET population from measurement of a di-labeled dsDNA sample. Figure S2 show a comparison of a FRET histogram obtained from the same burst with and without weights. The burst selection is obtained applying a burst size threshold of 10 counts (after background correction), in order to filter the extreme low-end of the burst size distribution.

The use of size-weighted FRET histograms is a simple way to obtain a representation of FRET distribution that maintains high power of resolving FRET peaks while including the full burst population and thus reducing statistical noise.

As a final remark, note that when increasing the size-threshold for burst selection the difference between weighted and unweighted FRET histograms tends to zero because the relative difference in burst weights in the selected burst becomes smaller (i.e. tends to 1, meaning equal weights).

References

[1] Antonino Ingargiola, Ted Laurence, Robert Boutelle, Shimon Weiss, and Xavier Michalet. Photon-HDF5: open data format and computational tools for timestamp-based single-molecule experiments. volume 971405. SPIE, 2016. `doi:10.1117/12.2212085`.

[2] Antonino Ingargiola, Ted Laurence, Robert Boutelle, Shimon Weiss, and Xavier Michalet. Photon-HDF5: An Open File Format for Timestamp-Based Single-Molecule Fluorescence Experiments. *Biophysical Journal*, 110(1):26–33, jan 2016. `doi:10.1016/j.bpj.2015.11.013`.

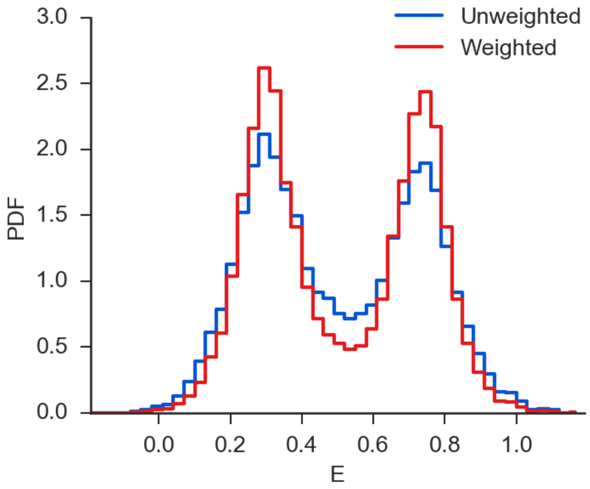

Figure S1: Comparison of unweighted and size-weighted FRET histograms for a simulated mixtures of static FRET populations. In both cases bursts are selected with a size threshold of 10 photons (after background correction).

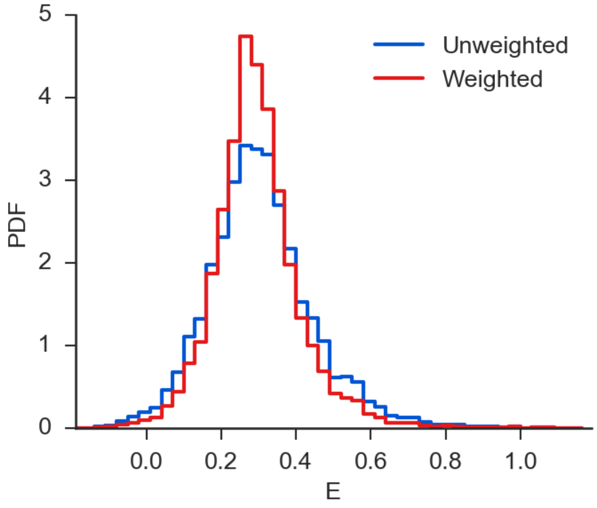

Figure S2: Comparison of unweighted and size-weighted FRET histograms for a smFRET measurement of a static FRET sample (di-labeled dsDNA). In both cases bursts are selected with a size threshold of 10 photons (after background correction).
